# Applicability of the Neuman Systems Model to the Gerontology Nursing practice: a scoping review

**DOI:** 10.1590/1518-8345.6977.4224

**Published:** 2024-07-29

**Authors:** Samara Gonçalves de Oliveira, Célia Pereira Caldas, Esther Mourão Nicoli, Frances Valéria Costa e Silva, Rosane Barreto Cardoso, Fernanda Maria do Vale Martins Lopes

**Affiliations:** 1 Universidade do Estado do Rio de Janeiro, Faculdade de Enfermagem, Rio de Janeiro, RJ, Brazil.; 2 Scholarship holder at the Fundação Carlos Chagas Filho de Amparo à Pesquisa do Estado do Rio de Janeiro (FAPERJ), Brazil.; 3 Universidade Federal do Rio de Janeiro, Faculdade de Enfermagem, Rio de Janeiro, RJ, Brazil.

**Keywords:** Aged, Health Services, Nursing, Nursing Theory, Nursing Care, Systems Theory

## Abstract

**Objective:**

to map scientific productions on the application of the Neuman Systems Model to the Nursing practice focused on health care for aged people.

**Method:**

a scoping review based on the methodology proposed by the Joanna Briggs Institute. Seven electronic databases were consulted. Regarding the eligibility criteria, the following were considered: Population - Aged people; Concept - Application of the Neuman Systems Model in the Nursing practice; and Research Context - Health services.

**Results:**

a total of 14 studies made up the sample. The data were analyzed and summarized into two categories: implementation of the Neuman Systems Model in hospital, institutional and outpatient settings; and use of the Neuman Systems Model in community and home environments.

**Conclusion:**

the application of Neuman Systems approach to the Gerontology Nursing practice, in different care scenarios, proved to be promising, considering aged people as comprehensive individuals with multiple dimensions. This perspective has shown adaptability and effectiveness in meeting the diverse needs of older adults, resulting in an improvement in their quality of life in old age.

## Highlights

**(1)** It is possible to integrate the systemic approach into the Gerontology Nursing practice.

**(2)** The Systems Model covers aged people’s needs in an integrative way.

**(3)** Actions to strengthen aged people’s lines of defense and resistance are important.

**(4)** The Model favors the identification of potential and real stressors for older adults.

**(5)** Studies with a systemic approach are required to promote healthy aging.

## Introduction

 The nurses’ role makes it possible to offer adequate support to each individual through the detailed identification of their care needs, as well as the development of actions that promote health in different care contexts and in all life cycles. Therefore, its action in assisting older adults can significantly contribute to improving the health and well-being of this population segment, considering population aging, a global phenomenon that has gained increasing visibility in the health sector in recent decades ^(^ ^1^ ^-^ ^2^ ^)^ . 

 The Neuman Systems Model is a theoretical approach that can be used to understand the behavior of aged people in relation to the health and disease process throughout life, considering aging. This approach is based on a holistic system that focuses the attention on the individual’s interaction with their unique environment, making it a valuable tool for nurses seeking to understand the complexities of aging ^(^ ^3^ ^-^ ^4^ ^)^ . 

 In the systemic view of this model, man and his environment are considered an open system, represented by concentric circles that include the nucleus, lines of resistance and lines of defense. Nursing interventions aim at preventing the stressors from penetrating the core and at helping restore lines of resistance and defense ^(^ ^3^ ^)^ . 

 The model postulates that health is a dynamic state of balance between a person and their environment and that defense barriers are broken when this balance is lost, which is expressed as illness. The goal of Nursing care is to help each person achieve and maintain adequate balance through appropriate interventions. The individual is perceived as an open system that interacts with internal or external environmental stressors. Due to each person’s historicity and dynamism, which are constantly changing due to reciprocal environmental interactions, not meeting their health needs reduces the subjective well-being condition ^(^ ^5^ ^)^ . 

 The conceptual breadth, flexibility and systemic properties of the Neuman Systems Model provide essential markers for an integrated view of leadership and scholarship, in accordance with Nursing philosophy, aiming to improve professional activities. As it is a holistic theoretical conception, its main characteristics are reliability, feasibility, flexibility and comprehensiveness. Such characteristics are fundamental to expand the professional roles and responsibilities of Nursing in the 21 ^st^ century ^(^ ^6^ ^)^ . 

From this perspective, the relevance of this scoping review stands out, aimed at reflecting on how the Neuman Systems Model is applied in the Gerontology Nursing context. This study is justified by its possibility of contributing to reflections on the implications of the Nursing care provided to older adults based on the Neuman Systems Model.

The objective of this research is to map scientific productions on the application of the Neuman Systems Model in the Nursing practice focused on health care for older adults.

## Method

 This is a scoping review guided by the recommendations set forth in the Joanna Briggs Institute (JBI) Reviewer’s Manual, carried out in nine stages: 1) Identification of the research question; 2) Identification of relevant studies; 3) Study selection; 4) Data mapping; 5) Compilation of the results; 6) Extraction of the evidence; 7) Analysis of the evidence; 8) Presentation of the results; and 9) Summary of all the evidence, conclusions and implications of the findings ^(^ ^7^ ^)^ . 

 The recommendations of the guidelines contained in the Preferred Reporting Items for Systematic reviews and Meta-Analyses extension for Scoping Reviews (PRISMA-ScR) checklist were followed ^(^ ^8^ ^)^ . 

 To construct the search strategy and research question, the “PCC” (Population, Concept and Context) mnemonic was used, recommended by JBI as a guide to create, in addition to the title and inclusion criteria, the research question ^(^ ^7^ ^,^ ^9^ ^)^ . 

The research question was formulated considering: Population – Aged people; Concept – Application of the Neuman Systems Model in the Nursing practice; and Research Context – Health services, structured as follows: How has the Neuman Systems Model been applied to the Nursing practice focused on health care for older adults?

 The search strategy was designed with the collaboration of two librarians, in order to identify relevant studies for this review. The objective was to analyze the controlled vocabulary and its synonyms, adapting the search syntax to each data source. Initially, a preliminary search was carried out on April 25 ^th^ , 2023 in the Medical Literature Analysis and Retrieval System Online (MEDLINE) databases via PubMed and in the Cumulative Index to Nursing & Allied Health Literature (CINAHL). This stage aimed at ensuring consistency in the study selection process, revealing a limited number of evidence sources on the topic proposed and absence of related reviews. 

### Protocol and registration

 The protocol for the scoping review was prepared and registered in the Open Science Framework (OSF), with the following DOI identifier: https://doi.org/10.17605/OSF.IO/9DCE6 . 

### Eligibility criteria

 The following eligibility criteria were considered: scientific papers that discussed how the theoretical perspective of the Neuman Systems Model is used in health care for aged people within the scope of health services; as well as studies that used the aged population as sample, defined as “people aged at least 60 years old”, according to the Older Adults’ Statute ^(^ ^10^ ^)^ . Scientific productions with heterogeneous sampling were excluded, that is, which included other age groups besides the elderly. 

Regarding the types of sources, studies with qualitative, quantitative and mixed-methods approaches were included. The incorporation of quantitative, qualitative and mixed-method studies aimed at capturing a diversified range of perspectives and evidence, providing a holistic analysis of the topic. Inclusion of these methodological approaches was intended to provide a more robust and contextualized understanding of the available knowledge about the applicability of the Neuman Systems Model in the Gerontology Nursing practice.

Studies unrelated to the topic, without any defined methodology, incomplete, reviews, editorials, book chapters, government documents (laws, decrees, ordinances), summaries, glossaries of scientific terms, annals, texts and opinion articles, letters, books, theses, dissertations and event summaries were excluded.

### Information sources

 The search for scientific productions was conducted on the ScienceDirect (Elsevier) and Web of Science academic literature platforms, in addition to the MEDLINE databases via PubMed, CINAHL, Embase, *Literatura Latino-Americana e do Caribe em Ciências da Saúde* (LILACS) and Scopus, without publication date restrictions. 

### Research

 The search for studies was conducted on May 29 ^th^ , whereas their selection took place in May and June 2023. 

### Selection of evidence sources

 The stages to select studies in the databases were: 1. Exporting the results obtained to a reference manager; 2. Removing duplicates; 3. Transferring files to the Rayyan Qatar Computing Research Institute (QCRI) software, which allows blinding in collaboration between reviewers ^(^ ^11^ ^)^ ; 4. Selecting studies according to the analysis of their titles and abstracts in an independent and double-blind way according to the pre-established analysis criteria for inclusion or exclusion of studies; 5. Solving disagreements in the selection process with a third reviewer; 6. Reading the full texts of the articles; and 7. Final selection of the texts included in the review. 

### Data mapping process

 To map the data, a search strategy was used with the ‘AND’ and ‘OR’ Boolean operators, incorporating controlled vocabularies and their synonyms. No filters related to language or time limits were applied, in order to ensure that all sources from the national and international literature were included. The search strategy outlined for the data sources is illustrated below in Figure 1 . 

**Figure 1:** - Search strategy developed, adapted to each data source. Rio de Janeiro, RJ, Brazil, 2023

**Data sources**	**Search strategy**	**Quantitative scientific productions**
**MEDLINE via PubMed**	(aged[MeSH] OR aged OR “aged patient” OR “aged subject” OR elderly OR “elderly population” OR “elderly patient” OR “elderly subject” OR senior OR “senior citizens” OR old OR “old-aged” OR “old people” OR “old person” OR “older people” OR “aged, 80 and over”[MeSH] OR “aged, 80 and over” OR “Oldest Old” OR “very elderly” OR “very old” OR “very elderly” OR eldest OR elder OR “middle aged”[MeSH] OR “middle aged”) AND (“neuman systems model” OR “neuman model” OR “neuman`s model” OR “neuman`s systems model”)	49
**CINAHL**	( (MH “Aged”) OR “aged” OR “elderly” OR ““elderly population”” OR “senior” OR ““senior citizens”” OR “old” OR ““old-aged”” OR ““old people”” OR ““older people”” OR “elder” OR “eldest” OR (MH “Aged, 80 and Over”) OR ““Aged, 80 and over”” OR ““Oldest Old”” OR (MH “Middle Age”) OR ““Middle Aged”” ) AND ( (MH “Neuman Systems Model”) OR “neuman systems model” OR ““neuman model”” OR ““neuman`s model”” OR ““neuman`s systems model”” )	40
**Embase**	(‘aged’/de OR ‘aged patient’ OR ‘aged people’ OR ‘aged person’ OR ‘aged subject’ OR ‘elderly’ OR ‘elderly patient’ OR ‘elderly people’ OR ‘elderly person’ OR ‘elderly subject’ OR ‘senior citizen’ OR ‘senior’ OR ‘senior citizens’ OR ‘aged’ OR ‘very elderly’/de OR ‘aged, 80 and over’ OR ‘very old’ OR ‘very elderly’ OR ‘middle aged’/de OR ‘middle age’ OR ‘middle aged’) AND (‘neuman systems model’/de OR ‘neuman model’ OR ‘neuman`s model’ OR ‘neuman`s systems model’ OR ‘neuman systems model’)	36
**Web of Science**	TS=((aged OR “aged patient” OR “aged subject” OR elderly OR “elderly population” OR “elderly patient” OR “elderly subject” OR senior OR “senior citizens” OR old OR “old-aged” OR “old people” OR “old person” OR “older people” OR “aged, 80 and over” OR “Oldest Old” OR “very elderly” OR “very old” OR “very elderly” OR eldest OR elder OR “middle aged” OR “middle age”) AND (“neuman systems model” OR “neuman model” OR “neuman`s model” OR “neuman`s systems model”))	20
**Scopus**	TITLE-ABS-KEY ( ( aged OR “aged patient” OR “aged subject” OR elderly OR “elderly population” OR “elderly patient” OR “elderly subject” OR senior OR “senior citizens” OR old OR “old-aged” OR “old people” OR “old person” OR “older people” OR “aged, 80 and over” OR “oldest old” OR “very elderly” OR “very old” OR “very elderly” OR eldest OR elder OR “middle aged” OR “Middle Age” ) AND ( “neuman systems model” OR “neuman model” OR “neuman`s model” OR “neuman`s systems model” ) )	59
**Science Direct**	(aged OR elderly OR “middle aged” OR “aged, 80 and over”) AND (“neuman systems model” OR “neuman model” OR “neuman`s model” OR “neuman`s systems model” OR “neuman systems model”)	112
**LILACS**	((mh:(“aged”) OR aged OR elderly OR “elderly population” OR senior OR “senior citizens” OR old OR “old-aged” OR “old people” OR “old person” OR “older people” OR elder OR eldest OR mh:(“aged, 80 and over”) OR “aged, 80 and over” OR “oldest old” OR mh:(“middle aged”) OR “middle aged”)) AND ((“neuman systems model” OR “neuman model” OR “neuman`s model” OR “neuman`s systems model”))	6

### Data items

 The diverse information from the texts included was organized and stored in an Excel ^®^ spreadsheet to ease extraction of the evidence. The following data items were considered: title; time (year when the study was published); authors; data source; locus (country of origin); purposes (objectives); main results; study population (requirements for participation in the study); study design (method); stress factors; effective strategies for coping with stressors; population (people aged at least 60 years old); application of the Systems Model to the Nursing practice; Nursing care possibilities; and context (health service in which Nursing care based on the Systems Model was provided). 

### Data treatment and analysis

In order to identify the essential components of each study, a descriptive analytical framework was adopted to critically examine the content of each selected paper.

The results were summarized by identifying the main themes addressed in the studies, allowing the analysis of content recurrence, convergence and divergence. This approach made it possible to understand applicability of the Neuman Systems Model to the Gerontology Nursing practice, resulting in the delineation of categories to discuss the findings.

### Ethical aspects

As this was research used secondary data, in the public domain and available in the literature, there was no need for ethical assessment. However, it is worth noting that copyright was respected with due citation and referencing of the studies.

## Results

**Figure 2:**
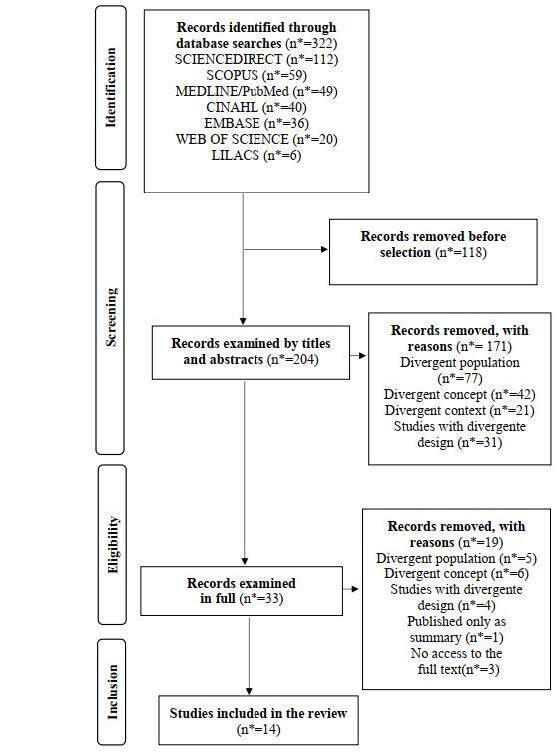
- Flowchart corresponding to the study selection process according to recommendations set forth in the Preferred Reporting Items for Systematic Review and Meta-Analysis extension for Scoping Reviews. Rio de Janeiro, RJ, Brazil, 2023 ^(^ ^8^ ^)^

 Initially, 322 relevant scientific productions were identified. A final sample of 14 studies was obtained. Figure 2 illustrates the path taken by the study selection process, from initial identification to creation of the final sample for analysis and discussion. 

 Based on their content, the studies were arranged into two categories: Implementation of the Neuman Systems Model in hospital, institutional and outpatient settings; and Use of the Neuman Systems Model in community and home environments. Figure 3 presents the characterization of the studies selected in the scoping review, according to country, year of publication, methodological approach and scientific journal. 

*n = Number of records

**Figure 3:** - Scientific productions selected according to the nature of the publication sources. Rio de Janeiro, RJ, Brazil, 2023

**Category 1 - Implementation of the Neuman Systems Model in hospital, institutional and outpatient settings**				
**Reference**	**Country/Year of Publication/ Data sources**	**Methodological approach/Type of study**	**Objectives**	**PCC** ^*^
Weinberger SL ^(^ ^12^ ^)^	United States/ 1991/ Rehabilitation Nursing	Qualitative/ Case report	To describe an analysis using the Neuman Systems Model to determine the effects of a colostomy on an 81-year-old client with cancer.	P ^†^: An 81-year-old person diagnosed with bowel cancer and using a colostomy bag. C ^‡^: The study explored the concepts of intrapersonal, interpersonal and extrapersonal stressors, in addition to psychological, sociocultural and physiological client variables, as well as lines of defense and secondary prevention actions. C ^§^: Hospital health service.
Potter ML, Zauszniewski JA ^(^ ^13^ ^)^ .	United States/ 2000/ Journal of Holistic Nursing	Quantitative/ Correlational and cross-sectional study	To examine variables that reflect reactions to stress, lines of defense and resistance and the basic core of human beings in the context of the Neuman Systems Model in a sample of aged people.	P ^†^: People aged at least 60 years old diagnosed with rheumatoid arthritis. C ^‡^: The study explored the concepts of client system variables (physiological, psychological, sociocultural, spiritual and developmental), lines of defense and resistance and the client’s basic core. C ^§^: Hospital health service.
Butts JB ^(^ ^14^ ^)^ .	United States/ 2001/ Geriatric Nursing	Quantitative/ Experimental study	To examine whether comforting touch improved perceptions of self-esteem, well-being and social processes, health status, life satisfaction and self-realization and faith or belief and self-responsibility of institutionalized aged women.	P ^†^: Woman aged at least 65 years old. C ^‡^: The study explored the concepts of client system variables (physiological, psychological, sociocultural, spiritual and developmental) to examine whether comforting touch improved perceptions of self-esteem, well-being and social processes, health status, life satisfaction and self-realization and faith or belief and self-responsibility. C ^§^: Two medium-sized nursing homes.
Lowry LW ^(^ ^15^ ^)^ .	United States/ 2012/ Nursing Science Quarterly	Qualitative/ Descriptive and interpretative	To explore the meaning of spirituality as described by older adults in various health states; to describe the relationship between spirituality and health; and to explain clients’ expectations for health professionals related to spirituality.	P ^†^: People aged at least 60 years old. C ^‡^: The study used the concept of the “spirituality” variable from the Neuman Systems Model. C ^§^: Nursing homes and Nursing care units.
Sousa JERB, Silva GRF, Luz MHBA, Pereira MLL ^(^ ^16^ ^)^ .	Spain/ 2015/ *Index de Enfermería*	Qualitative/ Case report	To show how the use of Neuman’s Theory associated with the prevention of pressure injuries employing the Braden Scale as a clinical standard is effective in reducing the incidence of pressure injuries in intensive care units.	P ^†^: A 94-year-old woman with acute respiratory failure, pneumonia and decompensated diabetes. C ^‡^: The study investigated client variables (physiological, psychological, sociocultural and developmental). In addition to that, it examined the nature and intensity of intrapersonal, interpersonal and extrapersonal stressors, considering their influence on the likelihood of these stressors emerging and reactions to them. It also analyzed the client’s interaction with environmental stressors, care strategies to maintain stability of the client system and achieve the highest possible level of overall client well-being. C ^§^: Hospital care service (Intensive Care Unit).
Rosa PH, Beuter M, Benetti ERR, Bruinsma JL, Venturini L, Backes C ^(^ ^17^ ^)^ .	Brazil/ 2018/ Anna Nery School Journal of Nursing	Qualitative/ Descriptive study	To describe the stressors experienced by hospitalized aged people from the perspective of the Neuman Systems Model	P ^†^: People aged at least 60 years old hospitalized for more than five days. C ^‡^: The study explored the concepts of intrapersonal, interpersonal and extrapersonal client system stressors. C ^§^: Hospital care service (Medical Clinic).
Benetti ERR, Beuter M, Rosa PH, Backes C, Jacobi CS, Oliveira FF ^(^ ^18^ ^)^ .	Brazil/ 2021/ *Revista de Enfermagem da Universidade Federal de Santa Maria*	Qualitative/ Convergent Care Research	To characterize hospitalized aged people according to the dimensions proposed by the Neuman Systems Model.	P ^†^: Hospitalized people aged at least 60 years old. C ^‡^: The study explored the Nursing Process based on this theoretical model, highlighting the concepts of: client variables (physiological, psychological, sociocultural and developmental); stressors; coping strategies related to stressors; and care actions to promote stability in the client’s system. C ^§^: Hospital care service (Medical Clinic I; Medical Clinic II and Surgical Clinic).
**Category 2 - Use of the Neuman Systems Model in community and home environments**				
**Reference**	**Country/Year of Publication**	**Methodological approach**	**Objectives**	**PCC** ^*^
Ross MM, Bourbonnais FF ^(^ ^19^ ^)^ .	United Kingdom/ 1985/ Journal of Advanced Nursing	Qualitative/ Case report	To describe how the Betty Neuman Systems Model is used in the practice employing a case report approach.	P ^†^: A 66-year-old person recovering at home after hospitalization and treatment for a myocardial infarction. C ^‡^: The study explored the concepts of intrapersonal, interpersonal and extrapersonal stressors, lines of defense and lines of client’s system resistance; in addition to primary, secondary and tertiary intervention strategies to promote stability in the client’s system. C ^§^: Home care service.
Ross MM, Helmer H ^(^ ^20^ ^)^ .	United States/ 1988/ Public Health Nursing	Qualitative/ Case report	To identify the similarities and differences between caring for an individual as a client and caring for a family as a client when using Betty Neuman’s Systems Model as a guide for the practice.	P ^†^: An aged couple (63-year-old woman and 66-year-old man). C ^‡^: The study explored the concepts of intrapersonal, interpersonal and extrapersonal stressors, lines of defense and lines of client’s system resistance; in addition to primary, secondary and tertiary intervention strategies to promote stability in the client’s system. C ^§^: Home care service.
Millard J ^(^ ^21^ ^)^ .	United Kingdom/ 1992/ British Journal of Nursing	Qualitative/ Case report	To analyze the role of health visitors in engaging with the elderly population, examining how assessment tools can influence the client’s perceptions about obtaining information and the care/support offered.	P ^†^: People aged at least 60 years old with Parkinson’s disease. C ^‡^: The study explored the concepts of intrapersonal, interpersonal and extrapersonal stressors, outlining short-, medium- and long-term care goals based on the client’s health needs through primary, secondary and tertiary prevention interventions. C ^§^: Community health service.
Imamura E ^(^ ^22^ ^)^ .	United States/ 2002/ International Journal of Nursing Practice	Qualitative/ Intervention study	To promote older adults’ understanding of chronic diseases; to inform them about proper nutrition and its impact on their health; to perform specific exercises for the elderly; and to ease building of supportive relationships among older adults.	P ^†^: People aged at least 65 years old. C ^‡^: A multidimensional questionnaire based on the concepts of client variables (physiological, psychological, sociocultural, spiritual and developmental) was developed to assess individual and behavioral experiences and explore the participants’ health conditions. C ^§^: Community health service.
Montano AR, Shellman J, Malcolm M, McDonald D, Rees C, Fortinsky R, Reagan L ^(^ ^23^ ^)^ .	United States/ 2020/ Geriatric Nursing	Quanti-qualitative/ Parallel mixed-methods convergent design	To evaluate the relationship between an Interprofessional Collaborative Practice (IPCP) intervention for community-dwelling older adults, Geriatric Outreach, and Training with Care! (GOT Care!), and reduced Emergency Department (ED) visits for the 51 aged participants.	P ^†^: People aged at least 65 years old living in the community identified as high risk, due to high use of emergency services or polypharmacy with multiple conditions. C ^‡^: The study used the concepts of client variables (physiological, psychological, sociocultural, spiritual and developmental), stressors, lines of defense, lines of resistance and primary, secondary and tertiary prevention strategies. C ^§^: Community health service.
Larijani F, Fotokian Z, Jahanshahi M, Tabi SR ^(^ ^24^ ^)^ .	Iran/ 2021/ Nursing and Midwifery Studies	Quantitative	To evaluate the effect of the Neuman Systems Model on the anxiety of aged people waiting for a colonoscopy.	P ^†^: People aged at least 60 years old fluent in Persian, who have not received anxiolytic or antipsychotic drugs in the last six months, no hearing or cognitive impairments, no history of severe stress in the last three months, no history of endoscopy or colonoscopy, and who achieved at least 50% of the total score on the Neuman Systems Model needs assessment checklist. C ^‡^: The study explored the concepts of client variables (physiological, psychological, sociocultural and developmental), potential and actual intrapersonal, interpersonal and extrapersonal stressors, goal setting, Nursing interventions and assessment, according to the Nursing Process supported by the Neuman Systems Model. C ^§^: Home health care.
Pereira F, Bieri M, Martins MM, Del Río Carral M, Verloo H ^(^ ^25^ ^)^ .	Italy/ 2022/ Nursing Reports	Qualitative/ Descriptive study	To identify and categorize the stressors experienced and the recovery strategies adopted by aged people, their informal caregivers and health professionals in the medication management of older adults after hospital discharge.	P ^†^: People aged at least 65 years old hospitalized in the last 90 days, managing at least five different medications daily. C ^‡^: The study explored the concepts of extrapersonal stressors and the extrapersonal reconstitution strategies adopted by aged people, their informal caregivers and health professionals in medication management after hospital discharge. C ^§^: Home health care.

* PCC = Population, Concept and Context;

† P = Population;

‡ C = Concept;

§ C = Context

Among the 14 studies selected, it was found that the prevalence of publications was higher in 2021, representing 14.2% (n=2) of the sample. On the other hand, the other 12 studies were distributed across different years of publication, with each year accounting for approximately 7.1% of the sample, represented by a single study (n=1).

Regarding the origin of the studies, it was highlighted that 50% (n=7) were from the United States of America (USA), whereas 14.2% (n=2) were carried out in Portugal and Brazil. The methodological approach that prevailed was qualitative (71.4% [n=10]), followed by quantitative with 21.4% (n=3) and quantitative-qualitative with 7.1% (n=1).

## Discussion

The studies selected were organized into two categories, considering the similarity of their content. The first category addresses the implementation of the Neuman Systems Model in hospital, institutional and outpatient care contexts; and the second category involves how the Neuman Systems Model is used in community and home contexts.

### Category 1 - Implementation of the Neuman Systems Model in hospital, institutional and outpatient settings

 Seven studies explored the applicability of the Neuman Systems Model in hospital, institutional and outpatient settings ^(^ ^12^ ^-^ ^18^ ^)^ . These research studies addressed specific conditions, such as: care for aged people with cancer assisted in the hospital care service ^(^ ^12^ ^)^ ; care for aged people with rheumatoid arthritis treated in a hospital care service ^(^ ^13^ ^)^ ; comforting touch practices in medium-sized nursing homes ^(^ ^14^ ^)^ ; encouraging spirituality in nursing homes and Nursing care units ^(^ ^25^ ^)^ ; prevention of pressure injuries in a hospital Intensive Care Unit ^(^ ^16^ ^)^ ; and identification and management of stressors during hospitalization, including medical and surgical clinics ^(^ ^17^ ^-^ ^18^ ^)^ . 

 The Systems Model approach proved to be effective in dealing with the stressors arising from the effects of colostomy in the care of aged women with cancer, covering different dimensions. At the intrapersonal level, the change in bowel habits triggered psychological stressors, resulting in emotional and psychological challenges that impacted the aged woman’s ability to adapt. In the interpersonal sphere, the absence of social support generated an additional negative impact, making the adaptation process even more difficult. In addition to that, hospitalization itself represented a stressor in the extrapersonal dimension, affecting the patient’s social interactions. The Nursing interventions were aimed at strengthening the aged woman’s lines of defense, providing adaptive support and seeking to maintain her optimal well-being ^(^ ^12^ ^)^ . 

 The need to explore in depth the beliefs and mechanisms for coping with cancer, evidenced in the study of the aged woman with a colostomy, is emphasized in the effectiveness of implementing the Neuman Systems Model in patients with rheumatoid arthritis. Applying this model to older adults with rheumatoid arthritis has proved to be effective in comprehensively considering the social, emotional and physical impacts of this chronic condition, incorporating elements such as spirituality and ability to adapt. When analyzing the combined effects of these impacts on aged people’s general perception of health, the importance of spirituality in coping with acute crises of the disease was highlighted. In addition to that, use of the model has fostered broader collaboration between patients and caregivers, offering relief and solutions to their complex health needs ^(^ ^13^ ^)^ . 

 There were also beneficial effects of the Neuman Systems Model, combined with comforting touch for self-esteem, well-being, social processes, health status, satisfaction with life, self-realization, faith or belief and self-responsibility of aged women assisted in long-term care institutions ^(^ ^14^ ^)^ . 

 The results of the study on comforting touch showed a significant impact on aged people’s perceptions, promoting improvements in well-being, self-esteem and other positively evaluated variables. These benefits are in line with the findings of the study that used the Systems Model to prevent pressure injuries in hospitalized patients, highlighting the effectiveness of approaches that consider not only physical factors but also emotional, social and environmental aspects to improve the patients’ health and well-being. In a way, the combined application of the Systems Model with the pressure injury risk assessment scale proved to be effective in primary prevention, allowing the Nursing team to adopt specific preventive measures to reduce potential stressors and strengthen the patients’ defenses against stress ^(^ ^16^ ^)^ . 

 In another research study, the relevance of health education to offer holistic care was highlighted, considering aged people’s spirituality based on the Neuman Model. Carried out in nursing homes and Nursing care units, the study revealed positive effects on mental and physical health, strengthening interpersonal relationships and the ability to face challenges. It was noticed that, as older adults’ health deteriorates, spiritual dependence increases, indicating a desire to receive spiritual care from health professionals ^(^ ^15^ ^)^ . 

 Using the Neuman Systems Model to identify and manage stressors during hospitalization of aged people also showed that certain stressors affect several clinical and personal areas, such as: advanced age; reduced physical capacity; limited knowledge and negative emotions; in addition to the influence exerted by family relationships, privacy, autonomy and the hospital environment itself ^(^ ^17^ ^)^ . Such findings have supported Nursing interventions focused on the Frail Elderly syndrome, sleep disorders, anxiety, fear and spiritual distress, aiming to mitigate the impact of these stressors and improve aged people’s experience during their hospitalization ^(^ ^18^ ^)^ . 

The findings of these studies highlight the efficacy of therapeutic interventions in hospital, institutional and outpatient settings in improving the patients’ self-esteem and well-being. The application of Neuman’s Systems Model revealed a significant impact on aged people’s quality of life, covering physical, psychological, social and spiritual dimensions. The spirituality approach proves to be a fundamental element in health interventions, influencing the perception of purpose in life and the ability to cope with stressors. The importance of interaction between aged people, family and health professionals is highlighted to promote affective bonds and trust, strengthening their lines of defense and resistance, resulting in effective interventions with a positive impact on their lives.

### Category 2 - Use of the Neuman Systems Model in community and home environments

 This category encompasses seven studies ^(^ ^19^ ^-^ ^25^ ^)^ , which explored how the Neuman Systems Model is applied in community and home environments, focusing on care to promote the aged people’s health through the following actions: home care for older adults after an acute myocardial infarction ^(^ ^19^ ^)^ ; post-hospital discharge home support after a stroke episode ^(^ ^20^ ^)^ ; care for aged people with Parkinson’s disease assisted by the community health service ^(^ ^21^ ^)^ ; health education activities carried out by the community health service ^(^ ^22^ ^)^ ; Interprofessional Collaborative Practice (IPCP) intervention for community-dwelling older adults ^(^ ^23^ ^)^ ; reduction of anxiety in aged people awaiting colonoscopy at home ^(^ ^24^ ^)^ ; and medication management after hospital discharge ^(^ ^25^ ^)^ . 

The Neuman Systems Model proved to be applicable in community and home environments, encompassing the family, the community and the home. It enabled a comprehensive assessment of the needs and resources of people and families alike, enabling the implementation of preventive, curative and rehabilitative interventions. In addition to that, the model valued the active participation of individuals and families in health care, strengthening the bond between health professionals and the community.

 This model has shown effectiveness in the home recovery of aged people after suffering an acute myocardial infarction, by promoting their well-being in line with their personal values, establishing progressive goals and appropriately using available resources ^(^ ^18^ ^)^ . 

 Thus, applying the Neuman Systems Model in tertiary prevention evidenced significant improvements in restoring balance in the client’s system after an acute myocardial infarction, reflecting similar results in a study on preventive actions aimed at the recovery of aged people after hospital discharge subsequent to a stroke episode. The study made it possible to identify individual and family stressors, such as: sleep deprivation; anxiety; communication difficulties; and lack of external support, with a care plan focused on promoting autonomy, optimizing sleep patterns and implementing environmental adjustments to ease recovery. These strategies included motivational, educational and behavioral interventions both at the individual and at the family context, highlighting the adaptability and effectiveness of the model in different clinical scenarios ^(^ ^19^ ^)^ . 

 The application of the Neuman Systems Model allowed nurses to establish effective care goals through primary, secondary and tertiary prevention interventions. These interventions were aligned with the short-, medium- and long-term health needs of an aged couple, in which one of the partners was diagnosed with Parkinson’s disease. These goals covered different aspects, such as emotional support, management of nocturnal incontinence and coping with aged people’s fears regarding loss of independence. Through efficient communication between the health professionals involved, it was possible to guarantee care continuity and offer the necessary support, resulting in an improvement in sleep quality for the aged man and a reduction in the social isolation of the wife, who played the caregiver role ^(^ ^20^ ^)^ . 

 Another crucial aspect was using the Neuman Systems Model as a solid longitudinal care tool through the community health service. Health education actions provided significant improvements in aged people’s well-being, equipping them with knowledge about preventive measures, encouraging changes in lifestyles and promoting the development of support networks. These impacts were especially notable among low-income older adults, resulting in the promotion of their health and improved quality of life ^(^ ^21^ ^)^ . 

 Thus, it is verified that implementing the Neuman Systems Model in community care plays a crucial role in aged people’s well-being. A study conducted in the community health service identified the contribution of stressors that impact older adults’ quality of life, followed by the development of primary, secondary and tertiary prevention strategies, in order to strengthen aged people’s lines of defense and resistance and promote their well-being ^(^ ^3^ ^)^ . 

 There were also positive results in reducing anxiety in older adults awaiting a colonoscopy at home, through specific interventions aimed at each stressor identified, such as bowel preparation, health problems, sleep disorders, fear of diagnosis, lack of knowledge and social isolation ^(^ ^24^ ^)^ . 

 Regarding medication management at home after aged people are discharged from the hospital, the identification of stressors related to this process highlighted problems regarding communication, collaboration and coordination of this care between older adults, informal caregivers and health professionals. Older adults’ non-participation in decisions about medication raised concerns, resulting in difficulties understanding the treatments and adhering to them. In this context, the importance of a patient-centered, interprofessional and collaborative approach to improving safety in medication management is highlighted ^(^ ^25^ ^)^ . 

The studies presented confirm the crucial role of Nursing in promoting older adults’ health, strengthening their support networks and implementing primary, secondary and tertiary prevention strategies in community and home contexts, aiming to maintain well-being and balance of the client’s system.

Regarding the knowledge gaps highlighted, there was lack of participatory, horizontal and intergenerational proposals for effectively coping with stressors throughout life considering healthy aging. In addition to that, the study highlighted the scarcity of surveys into the role of nurses as members of multidisciplinary teams, focusing on the identification of stressors and the implementation of appropriate interventions in different care contexts, aiming to promote healthy aging and effective coping with stressors throughout life.

This study faced some limitations, including the fact that three full publications were not accessible, restricting the possibility of analyzing these documents in depth. Additionally, other limitations were evidenced. The search strategy employed may not have covered all the relevant terms, particularly considering the “health services” context, as this term presents a wide terminological variation between different nations. Expanding the review to other relevant databases might also have expanded the study scope, allowing the incorporation of additional research studies on the topic proposed.

## Conclusion

It was verified that integrating the Neuman Systems approach in the Gerontology Nursing practice, in different care settings such as hospital, institutional, outpatient, community and home environments, revealed efficacy and adaptability when considering aged people as comprehensive and multidimensional individuals.

This approach meets the diverse needs of older adults by considering the client’s five variables (physiological, psychological, spiritual, sociocultural and development), implementing care actions to strengthen the client’s lines of defense and resistance, in addition to recognizing the client’s potential and real stressors (intrapersonal, interpersonal and extrapersonal), using primary, secondary and tertiary intervention strategies to improve their well-being and quality of life.

Development of this research implies developing studies aimed at promoting healthy aging, considering the conceptual elements of the theory and the complexities inherent to senescence, with the aim of improving Nursing practices to benefit the well-being of the aging population.
