# Supplementary material for: Applicability of the Neuman Systems Model to the Gerontology Nursing practice: a scoping review
Source: Rev Lat Am Enfermagem. 2024 Jul 29;32:e4224. doi: 10.1590/1518-8345.6977.4224 (PMC11295264; doi:10.1590/1518-8345.6977.4224)
Supplement: Translation

# Translation: Aplicabilidad del Modelo de Sistemas de Neuman a la práctica de enfermería gerontológica: revisión de alcance

**DOI:** 10.1590/1518-8345.6977.4223

**Authors:** Samara Gonçalves de Oliveira, Célia Pereira Caldas, Esther Mourão Nicoli, Frances Valéria Costa e Silva, Rosane Barreto Cardoso, Fernanda Maria do Vale Martins Lopes

**Affiliations:** 1 Universidade do Estado do Rio de Janeiro, Faculdade de Enfermagem, Rio de Janeiro, RJ, Brasil.; 2 Becaria de la Fundação Carlos Chagas Filho de Amparo à Pesquisa do Estado do Rio de Janeiro (FAPERJ), Brasil.; 3 Universidade Federal do Rio de Janeiro, Faculdade de Enfermagem, Rio de Janeiro, RJ, Brasil.

**Keywords:** Anciano, Servicios de Salud, Enfermería, Teoría de Enfermería, Atención de Enfermería, Teoría de Sistemas

## Abstract

**Objetivo:**

mapear producciones científicas sobre la aplicación del Modelo de Sistemas de Neuman a la práctica de enfermería para la atención de la salud del adulto mayor.

**Método:**

revisión de alcance, basada en la metodología propuesta por el *Joanna Briggs Institute* . Se consultaron siete bases de datos electrónicas. Se consideraron los siguientes criterios de elegibilidad: Población - adultos mayores; Concepto - aplicación del Modelo de Sistemas de Neuman a la práctica de enfermería; y Contexto de la investigación - servicios de salud.

**Resultados:**

la muestra estuvo compuesta por 14 estudios. Los datos se analizaron y resumieron en dos categorías: implementación del Modelo de Sistemas de Neuman en ambientes hospitalarios, institucionales y ambulatorios y uso del Modelo de Sistemas de Neuman en el ambiente comunitario y el domiciliario.

**Conclusión:**

la aplicación del enfoque sistémico de Neuman a la práctica de enfermería gerontológica, en diferentes escenarios de atención, resultó prometedora, al considerar al adulto mayor como individuo integral y con múltiples dimensiones. Se comprobó que esa perspectiva cuenta con adaptabilidad y eficacia para satisfacer las diferentes necesidades de los adultos mayores, y que mejora la calidad de vida en la vejez.

## Destacados

**(1)** Se puede integrar el enfoque sistémico a la práctica de enfermería gerontológica.

**(2)** El Modelo de Sistemas satisface las necesidades de los adultos mayores de forma integral.

**(3)** Es importante que se realicen acciones para fortalecer las líneas de defensa y resistencia de los adultos mayores.

**(4)** El Modelo permite identificar factores estresores potenciales y reales para los adultos mayores.

**(5)** Es necesario que se realicen estudios con enfoque sistémico para promover el envejecimiento saludable.

## Introducción

 El enfermero puede brindarle el apoyo adecuado al individuo, a través de la identificación detallada de sus necesidades de cuidado y del desarrollo de acciones que promuevan la salud en diferentes contextos de atención y en todos los ciclos de vida. Por lo tanto, su participación en la atención de los adultos mayores puede contribuir significativamente a mejorar la salud y el bienestar de esta población, considerando el envejecimiento poblacional, fenómeno global que ha ganado cada vez más visibilidad en el sector de la salud en las últimas décadas ^(^ ^1^ ^-^ ^2^ ^)^ . 

 El Modelo de Sistemas de Neuman es un enfoque teórico que puede utilizarse para comprender el comportamiento de los adultos mayores en lo que respecta al proceso de salud y enfermedad durante toda la vida, considerando el envejecimiento. Este enfoque se basa en un sistema holístico que enfoca la atención en la interacción del individuo con su ambiente específico, y es una herramienta valiosa para los enfermeros que buscan comprender las complejidades del envejecimiento ^(^ ^3^ ^-^ ^4^ ^)^ . 

 En la visión sistémica de este modelo, se considera que el hombre y su entorno son sistemas abiertos, representados por círculos concéntricos que incluyen el núcleo, las líneas de resistencia y las líneas de defensa. El objetivo de las intervenciones de enfermería es prevenir que los factores estresores penetren en el núcleo y ayudar a restaurar las líneas de resistencia y defensa ^(^ ^3^ ^)^ . 

 El modelo postula que la salud es un estado dinámico de equilibrio entre el individuo y su entorno y que cuando se pierde ese equilibrio se rompen las barreras de defensa, lo que se manifiesta como enfermedad. El objetivo de los cuidados de enfermería es ayudar al individuo a alcanzar y mantener un equilibrio adecuado mediante intervenciones apropiadas. Se considera al individuo como un sistema abierto interactivo que interactúa con factores estresores ambientales internos o externos. Debido a la historicidad y el dinamismo de cada individuo, que cambia constantemente a causa de la interacción ambiental recíproca, cuando no se satisfacen sus necesidades de salud se reduce la condición de bienestar subjetivo ^(^ ^5^ ^)^ . 

 La amplitud conceptual, la flexibilidad y las propiedades sistémicas del Modelo de Sistemas de Neuman proporcionan marcadores esenciales para tener una visión integral de liderazgo y erudición, que coincide con la filosofía de la enfermería, con el objetivo de mejorar la actividad profesional. Al ser una concepción teórica holística, sus principales características son confiabilidad, viabilidad, flexibilidad y exhaustividad. Dichas características son fundamentales para ampliar los roles y las responsabilidades profesionales de los enfermeros en el siglo XXI ^(^ ^6^ ^)^ . 

Por lo tanto, se destaca la relevancia que tiene esta revisión de alcance, cuyo objetivo es reflexionar sobre la aplicación del Modelo de Sistemas de Neuman en el contexto de la enfermería gerontológica. Este estudio es importante porque puede contribuir a la reflexión sobre las implicancias que tiene la atención de enfermería que se le brinda al adulto mayor basada en el Modelo de Sistemas de Neuman.

El objetivo de esta investigación es mapear producciones científicas sobre la aplicación del Modelo de Sistemas de Neuman a la práctica de enfermería centrada en la atención de la salud del adulto mayor.

## Método

 Esta es una revisión de alcance que sigue las recomendaciones del *Joanna Briggs Institute Reviewer’s Manual* (JBI), y se realizó en nueve etapas: 1) identificación de la pregunta de investigación; 2) identificación de estudios relevantes; 3) selección de estudios; 4) mapeo de datos; 5) recopilación de resultados; 6) extracción de evidencia; 7) análisis de evidencia; 8) presentación de resultados; y 9) resumen de la evidencia, conclusiones e implicancias de los hallazgos ^(^ ^7^ ^)^ . 

 Se siguieron las recomendaciones de las pautas presentes en la *checklist* del *Preferred Reporting Items for Systematic reviews and Meta-Analyses extension for Scoping Reviews* (PRISMA-ScR) ^(^ ^8^ ^)^ . 

 Para elaborar la estrategia de búsqueda y la pregunta de investigación se utilizó el nemotécnico “PCC” (población, concepto y contexto), recomendado por el JBI como guía para elaborar, además del título y los criterios de inclusión, la pregunta de investigación ^(^ ^7^ ^,^ ^9^ ^)^ . 

La pregunta de investigación fue formulada considerando: Población - adultos mayores; Concepto - aplicación del Modelo de Sistemas de Neuman a la práctica de enfermería; y Contexto de investigación - servicios de salud, estructurada de la siguiente manera: ¿Cómo se aplicó el Modelo de Sistemas de Neuman a la práctica de enfermería centrada en la atención de la salud del adulto mayor?

 La estrategia de búsqueda se diseñó con la colaboración de dos bibliotecarias para identificar estudios relevantes para esta revisión. El objetivo fue analizar el vocabulario controlado y sus sinónimos, adaptando la sintaxis de búsqueda a cada fuente de datos. Inicialmente, se realizó una búsqueda preliminar el 25 de abril de 2023 en las bases de datos *Medical Literature Analysis and Retrieval System Online* (MEDLINE) a través de PubMed y *Cumulative Index to Nursing & Allied Health Literature* (CINAHL). El objetivo de esta etapa fue garantizar la coherencia en el proceso de selección de estudios, que reveló un número limitado de evidencia sobre el tema propuesto y la ausencia de revisiones relacionadas. 

### Protocolo y registro

 Se elaboró el protocolo de revisión de alcance, registrado en el *Open Science Framework* (OSF), con el siguiente identificador DOI: https://doi.org/10.17605/OSF.IO/9DCE6 . 

### Criterios de elegibilidad

 Como criterios de elegibilidad se consideraron: trabajos científicos que discutieran el uso de la perspectiva teórica del Modelo de Sistemas de Neuman en la atención a la salud de los adultos mayores en el ámbito de los servicios de salud; y estudios cuya muestra poblacional fuesen adultos mayores, definida como “personas de 60 años o más”, según el Estatuto del Adulto Mayor ^(^ ^10^ ^)^ . Se excluyeron las producciones científicas con muestreo heterogéneo, es decir, que incluyeran otros grupos etarios además de los adultos mayores. 

En cuanto a los tipos de fuentes, se incluyeron estudios con enfoque cualitativo, cuantitativo y de método mixto. La incorporación de estudios cuantitativos, cualitativos y de métodos mixtos tuvo como objetivo incluir una amplia gama de perspectivas y evidencia, que permiten realizar un análisis holístico del tema. Al incluir estos enfoques metodológicos se obtiene una comprensión más sólida y contextualizada del conocimiento disponible sobre la aplicabilidad del Modelo de Sistemas de Neuman en la práctica de enfermería gerontológica.

Se excluyeron los estudios que no se relacionaban con al tema, no tenían una metodología definida, estaban incompletos, los estudios de revisión, editoriales, capítulos de libros, documentos gubernamentales (leyes, decretos, ordenanzas), reseñas, glosarios de términos científicos, anales, textos y artículos de opinión, cartas, libros, tesis, disertaciones y resúmenes de eventos.

### Fuentes de información

 La búsqueda de producciones científicas se realizó en las plataformas de literatura académica *ScienceDirect* (Elsevier) y *Web of Science* , y en las bases de datos MEDLINE a través de PubMed, CINAHL, Embase, Literatura Latinoamericana y del Caribe en Ciencias de la Salud (LILACS) y Scopus, sin límite de fecha de publicación. 

### Investigación

La búsqueda de estudios se realizó el 29 de mayo y la selección se realizó en mayo y junio de 2023.

### Selección de fuentes de evidencia

 Las etapas de selección de estudios en las bases de datos fueron: 1. Exportación de los resultados obtenidos a un gestor de referencias; 2. Eliminación de duplicados; 3. Transferencia de archivos al *software Rayyan Qatar Computing Research Institute* (QCRI), que permite el cegamiento en la colaboración entre los revisores ^(^ ^11^ ^)^ ; 4. Selección de estudios según el análisis del título y resumen de forma independiente y doble ciego según los criterios de análisis preestablecidos para la inclusión o exclusión de estudios; 5. Resolución de discrepancias en el proceso de selección por medio de un tercer revisor; 6. Lectura de los textos completos de los artículos; y 7. Selección final de los textos incluidos en la revisión. 

### Proceso de mapeo de datos

 Para mapear los datos se utilizó una estrategia de búsqueda con los operadores booleanos ‘AND’ y ‘OR’, incorporando vocabularios controlados y sus sinónimos. Para garantizar la inclusión de todas las fuentes de literatura nacional e internacional no se aplicaron filtros relacionados con el idioma o el límite de tiempo. La estrategia de búsqueda descrita para las fuentes de datos se ilustra a continuación en la Figura 1 . 

**Figura 1:** - Estrategia de búsqueda realizada, adaptada a cada fuente de datos. Río de Janeiro, RJ, Brasil, 2023

**Fuentes de datos**	**Estrategia de Búsqueda**	**Número de producciones científicas**
**MEDLINE a través PubMed**	(aged[MeSH] OR aged OR “aged patient” OR “aged subject” OR elderly OR “elderly population” OR “elderly patient” OR “elderly subject” OR senior OR “senior citizens” OR old OR “old-aged” OR “old people” OR “old person” OR “older people” OR “aged, 80 and over”[MeSH] OR “aged, 80 and over” OR “Oldest Old” OR “very elderly” OR “very old” OR “very elderly” OR eldest OR elder OR “middle aged”[MeSH] OR “middle aged”) AND (“neuman systems model” OR “neuman model” OR “neuman`s model” OR “neuman`s systems model”)	49
**CINAHL**	( (MH “Aged”) OR “aged” OR “elderly” OR ““elderly population”” OR “senior” OR ““senior citizens”” OR “old” OR ““old-aged”” OR ““old people”” OR ““older people”” OR “elder” OR “eldest” OR (MH “Aged, 80 and Over”) OR ““Aged, 80 and over”” OR ““Oldest Old”” OR (MH “Middle Age”) OR ““Middle Aged”” ) AND ( (MH “Neuman Systems Model”) OR “neuman systems model” OR ““neuman model”” OR ““neuman`s model”” OR ““neuman`s systems model”” )	40
**Embase**	(‘aged’/de OR ‘aged patient’ OR ‘aged people’ OR ‘aged person’ OR ‘aged subject’ OR ‘elderly’ OR ‘elderly patient’ OR ‘elderly people’ OR ‘elderly person’ OR ‘elderly subject’ OR ‘senior citizen’ OR ‘senior’ OR ‘senior citizens’ OR ‘aged’ OR ‘very elderly’/de OR ‘aged, 80 and over’ OR ‘very old’ OR ‘very elderly’ OR ‘middle aged’/de OR ‘middle age’ OR ‘middle aged’) AND (‘neuman systems model’/de OR ‘neuman model’ OR ‘neuman`s model’ OR ‘neuman`s systems model’ OR ‘neuman systems model’)	36
***Web of Science***	TS=((aged OR “aged patient” OR “aged subject” OR elderly OR “elderly population” OR “elderly patient” OR “elderly subject” OR senior OR “senior citizens” OR old OR “old-aged” OR “old people” OR “old person” OR “older people” OR “aged, 80 and over” OR “Oldest Old” OR “very elderly” OR “very old” OR “very elderly” OR eldest OR elder OR “middle aged” OR “middle age”) AND (“neuman systems model” OR “neuman model” OR “neuman`s model” OR “neuman`s systems model”))	20
**Scopus**	TITLE-ABS-KEY ( ( aged OR “aged patient” OR “aged subject” OR elderly OR “elderly population” OR “elderly patient” OR “elderly subject” OR senior OR “senior citizens” OR old OR “old-aged” OR “old people” OR “old person” OR “older people” OR “aged, 80 and over” OR “oldest old” OR “very elderly” OR “very old” OR “very elderly” OR eldest OR elder OR “middle aged” OR “Middle Age” ) AND ( “neuman systems model” OR “neuman model” OR “neuman`s model” OR “neuman`s systems model” ) )	59
***Science Direct***	(aged OR elderly OR “middle aged” OR “aged, 80 and over”) AND (“neuman systems model” OR “neuman model” OR “neuman`s model” OR “neuman`s systems model” OR “neuman systems model”)	112
**LILACS**	((mh:(“aged”) OR aged OR elderly OR “elderly population” OR senior OR “senior citizens” OR old OR “old-aged” OR “old people” OR “old person” OR “older people” OR elder OR eldest OR mh:(“aged, 80 and over”) OR “aged, 80 and over” OR “oldest old” OR mh:(“middle aged”) OR “middle aged”)) AND ((“neuman systems model” OR “neuman model” OR “neuman`s model” OR “neuman`s systems model”))	6

### Ítems de datos

 La información de los textos incluidos se organizó y almacenó en una hoja de cálculo en el programa Excel ^®^ para facilitar la extracción de evidencia. Se consideraron los siguientes ítems de datos: título; tiempo (año de publicación); autores; fuente de datos; ubicación (país de origen); propósitos (objetivos); resultados principales; población de estudio (requisitos para participar en el estudio); diseño del estudio (método); factores estresores; estrategias efectivas para afrontar los factores estresores; población (personas de 60 años o más); aplicación del Modelo de Sistemas a la práctica de enfermería; posibilidades de atención de enfermería; y contexto (servicio de salud en el que se brindó atención de enfermería en base al Modelo de Sistemas). 

### Procesamiento y análisis de datos

Para identificar los componentes esenciales de cada estudio, se adoptó un marco analítico descriptivo para realizar un análisis crítico del contenido de cada investigación seleccionada.

El resumen de los resultados se realizó identificando los principales temas abordados en los estudios, que permiten analizar la recurrencia, convergencia y divergencia del contenido. Este abordaje permitió comprender la aplicabilidad del Modelo de Sistemas de Neuman a la práctica de enfermería gerontológica, y establecer categorías para discutir los hallazgos.

### Aspectos éticos

Como se trataba de una investigación que utilizó datos secundarios, de dominio público y disponibles en la literatura, no fue necesario someterla a evaluación ética. Sin embargo, cabe destacar que se respetaron los derechos de autor al realizar la correcta citación e incluir las referencias de los estudios.

## Resultados

 Inicialmente se identificaron 322 producciones científicas relevantes. Se obtuvo una muestra final de 14 estudios. La Figura 2 ilustra el camino del proceso de selección de los estudios, desde la identificación inicial hasta la elaboración de la muestra final para su análisis y discusión. 

 En función del contenido, los estudios se organizaron en dos categorías: implementación del Modelo de Sistemas de Neuman en ambientes hospitalarios, institucionales y ambulatorios; y uso del Modelo de Sistemas de Neuman en el ambiente comunitario y el domiciliario. La Figura 3 presenta la caracterización de los estudios seleccionados en la revisión de alcance, según el país, año de publicación, enfoque metodológico y revista científica. 

**Figura 2:** - Diagrama de flujo de la selección de los estudios según las recomendaciones del *Preferred Reporting Items for Systematic Review and Meta-Analysis extension for Scoping Reviews* . Río de Janeiro, RJ, Brasil, 2023 ^(^ ^8^ ^)^

*n = Número de registros

**Figura 3:** - Producciones científicas seleccionadas según la naturaleza de las fuentes de publicación. Río de Janeiro, RJ, Brasil, 2023

**Categoría 1 - Implementación del Modelo de Sistemas de Neuman en ambientes hospitalarios, institucionales y ambulatorios**				
**Referencia**	**País/Año de Publicación/ Fuente de datos**	**Enfoque metodológico/ Tipo de estudio**	**Objetivos**	**PCC** ^*^
Weinberger SL ^(^ ^12^ ^)^ .	Estados Unidos/ 1991/ *Rehabilitation Nursing*	Cualitativo/ Informe de caso	Describir un análisis utilizando el Modelo de Sistemas de Neuman para determinar los efectos de una colostomía en un paciente de 81 años con cáncer.	P ^†^: Persona de 81 años diagnosticada con cáncer de intestino que usa una bolsa de colostomía. C ^‡^: El estudio exploró los conceptos de estresores intrapersonales, interpersonales, extrapersonales, las variables psicológicas, socioculturales y fisiológicas del paciente, así como las líneas de defensa y acciones de prevención secundaria. C ^§^: Servicio de salud hospitalario.
Potter ML, Zauszniewski JA ^(^ ^13^ ^)^ .	Estados Unidos/ 2000/ *Journal of Holistic Nursing*	Cuantitativo/ Estudio correlacional y transversal	Analizar variables que reflejan reacciones al estrés, líneas de defensa y resistencia y el núcleo básico del ser humano en el contexto del Modelo de Sistemas de Neuman en una muestra de adultos mayores.	P ^†^: Personas de 60 años o más diagnosticadas con artritis reumatoide. C ^‡^: El estudio exploró los conceptos de variables del sistema paciente (fisiológicas, psicológicas, socioculturales, espirituales y de desarrollo), líneas de defensa y resistencia y el núcleo básico del paciente. C ^§^: Servicio de salud hospitalario.
Butts JB ^(^ ^14^ ^)^ .	Estados Unidos/ 2001/ *Geriatric Nursing*	Cuantitativo/ Estudio experimental	Analizar si el confort mejoraba las percepciones de autoestima, bienestar y procesos sociales, estado de salud, satisfacción con la vida y autorrealización y fe o creencia y autorresponsabilidad de mujeres mayores institucionalizadas.	P ^†^: Mujer de 65 años o más. C ^‡^: El estudio exploró los conceptos de variables del sistema paciente (fisiológicas, psicológicas, socioculturales, espirituales y de desarrollo) para examinar si el confort mejoraba las percepciones de autoestima, bienestar y procesos sociales, estado de salud, satisfacción con la vida y autorrealización y fe o creencia y autorresponsabilidad. C ^§^: Dos residencias para personas mayores de tamaño medio.
Lowry LW ^(^ ^15^ ^)^ .	Estados Unidos/ 2012/ *Nursing Science Quarterly*	Cualitativo/ Descriptivo e interpretativo	Explorar el significado de la espiritualidad tal como la describen los adultos mayores en diversos estados de salud, describir la relación entre la espiritualidad y la salud y explicar las expectativas de los pacientes sobre los profesionales de la salud relacionadas con la espiritualidad.	P ^†^: Persona de 60 años o más. C ^‡^: El estudio utilizó el concepto de variable espiritualidad del Modelo de Sistemas de Neuman. C ^§^: Residencias para personas mayores y unidades de atención de enfermería.
Sousa JERB, Silva GRF, Luz MHBA, Pereira MLL ^(^ ^16^ ^)^ .	España/ 2015/ Index de Enfermería	Cualitativo/ Informe de caso	Mostrar cómo el uso de la Teoría de Neuman asociada a la prevención de lesiones por presión utilizando la Escala de Braden como estándar clínico es eficaz para reducir la incidencia de lesiones por presión en la unidad de cuidados intensivos.	P ^†^: Mujer de 94 años con insuficiencia respiratoria aguda, neumonía y diabetes descompensada. C ^‡^: El estudio investigó variables del paciente (fisiológicas, psicológicas, socioculturales y de desarrollo). Además, analizó la naturaleza y la intensidad de los estresores intrapersonales, interpersonales y extrapersonales, considerando su influencia en la probabilidad de aparición y reacción a estos estresores. También se analizó la interacción del paciente con factores estresores ambientales, estrategias de atención para mantener la estabilidad del sistema paciente y lograr el nivel más alto posible de bienestar general del paciente. C ^§^: Servicio de atención hospitalaria (unidad de cuidados intensivos).
Rosa PH, Beuter M, Benetti ERR, Bruinsma JL, Venturini L, Backes C ^(^ ^17^ ^)^ .	Brasil/ 2018/ *Escola Anna Nery Revista de Enfermagem*	Cualitativo/ Estudio descriptivo	Describir los estresores que experimentan las personas mayores hospitalizadas desde la perspectiva del Modelo de Sistemas de Neuman.	P ^†^: Persona de 60 años o más hospitalizadas hace más de cinco días. C ^‡^: El estudio exploró los conceptos de factores estresores intrapersonales, interpersonales y extrapersonales del sistema paciente. C ^§^: Servicio de atención hospitalaria (clínica médica).
Benetti ERR, Beuter M, Rosa PH, Backes C, Jacobi CS, Oliveira FF ^(^ ^18^ ^)^ .	Brasil/ 2021/ *Revista de Enfermagem da Universidade Federal de Santa Maria*	Cualitativo/ Investigación Convergente Asistencial	Caracterizar a los adultos mayores hospitalizados según las dimensiones propuestas por el Modelo de Sistemas de Neuman.	P ^†^: Personas de 60 años o más hospitalizadas. C ^‡^: El estudio analizó el Proceso de Enfermería siguiendo este modelo teórico, destacando los conceptos de: variables del paciente (fisiológicas, psicológicas, socioculturales y de desarrollo), estresores, estrategias de afrontamiento relacionadas con los estresores y acciones de cuidado para promover la estabilidad del paciente. C ^§^: Servicio de atención hospitalaria (Clínica Médica I; Clínica Médica II y Clínica Quirúrgica).
**Categoría 2 - Uso del Modelo de Sistemas de Neuman en el ambiente comunitario y el domiciliario**				
**Referencia**	**País/ Año de Publicación/ Fuente de datos**	**Enfoque metodológico**	**Objetivos**	**PCC** ^*^
Ross MM, Bourbonnais FF ^(^ ^19^ ^)^ .	Reino Unido/ 1985/ *Journal of Advanced Nursing*	Cualitativo/ Informe de caso.	Describir el uso del Modelo de Sistemas Betty Neuman en la práctica utilizando un enfoque de Informe de caso.	P ^†^: Persona de 66 años en proceso de recuperación domiciliaria tras hospitalización y tratamiento por infarto de miocardio. C ^‡^: El estudio exploró los conceptos de estresores intrapersonales, interpersonales y extrapersonales, líneas de defensa, líneas de resistencia del sistema paciente y estrategias de intervención primaria, secundaria y terciaria para promover la estabilidad del sistema paciente. C ^§^: Servicio de atención domiciliaria.
Ross MM, Helmer H ^(^ ^20^ ^)^ .	Estados Unidos/ 1988/ *Public Health Nursing*	Cualitativo/ Informe de caso.	Identificar las semejanzas y diferencias entre cuidar a un individuo como paciente y cuidar a una familia como paciente al utilizar el modelo de sistemas de Betty Neuman como guía para la práctica.	P ^†^: Una pareja de adultos mayores (la mujer de 63 años y el hombre de 66 años). C ^‡^: El estudio exploró los conceptos de estresores intrapersonales, interpersonales y extrapersonales, líneas de defensa, líneas de resistencia del sistema paciente y estrategias de intervención primaria, secundaria y terciaria para promover la estabilidad del sistema paciente. C ^§^: Servicio de atención domiciliaria.
Millard J ^(^ ^21^ ^)^ .	Reino Unido/ 1992/ *British Journal of Nursing*	Cualitativo/ Informe de caso	Analizar el papel de los visitadores sanitarios en la interacción con la población de edad avanzada, examinando cómo las herramientas de evaluación pueden influir en la percepción del paciente sobre la obtención de información y la atención/apoyo brindado.	P ^†^: Persona de 60 años o más con Parkinson. C ^‡^: El estudio exploró los conceptos de estresores intrapersonales, interpersonales y extrapersonales, delineó objetivos de atención a corto, mediano y largo plazo basados en las necesidades de salud del paciente a través de intervenciones de prevención primaria, secundaria y terciaria. C ^§^: Servicio de salud comunitario.
Imamura E ^(^ ^22^ ^)^ .	Estados Unidos/ 2002/ *International Journal of Nursing Practice*	Cualitativo/ Estudio de Intervención	Promover la comprensión de las personas mayores sobre las enfermedades crónicas; brindarles información sobre nutrición adecuada y el impacto que tiene en su salud; realizar ejercicios específicos para personas mayores; y facilitar la construcción de relaciones de apoyo entre los adultos mayores.	P ^†^: Personas de 65 años o más. C ^‡^: Se desarrolló un cuestionario multidimensional, basado en los conceptos de variables del paciente (fisiológicas, psicológicas, socioculturales, espirituales y de desarrollo), para evaluar las experiencias individuales y conductuales y analizar las condiciones de salud de los participantes. C ^§^: Servicio de salud comunitario.
Montano AR, Shellman J, Malcolm M, McDonald D, Rees C, Fortinsky R, Reagan L ^(^ ^23^ ^)^ .	Estados Unidos/ 2020/ *Geriatric Nursing*	Cuantitativo y Cualitativo/ Proyecto convergente de métodos mixtos paralelos	Evaluar la relación entre una intervención de práctica colaborativa interprofesional (IPCP) para adultos mayores que viven en la comunidad, ¡ *Geriatric Outreach and Training with Care!* ( *GOT Care!* ), y la reducción de visitas al Departamento de Emergencias (DE) para las 51 personas mayores participantes.	P ^†^: Personas de 65 años o más residentes en la comunidad identificadas como de alto riesgo, debido al alto uso de servicios de emergencia o polifarmacia con múltiples condiciones. C ^‡^: El estudio utilizó los conceptos de variables del paciente (fisiológicas, psicológicas, socioculturales, espirituales y de desarrollo), estresores, líneas de defensa, líneas de resistencia y estrategias de prevención primaria, secundaria y terciaria. C ^§^: Servicio de salud comunitario.
Larijani F, Fotokian Z, Jahanshahi M, Tabi SR ^(^ ^24^ ^)^ .	Irán/ 2021/ *Nursing and Midwifery Studies*	Cuantitativo	Evaluar el efecto del modelo de sistemas de Neuman sobre la ansiedad de personas mayores a la espera de una colonoscopia.	P ^†^: Personas de 60 años o más que hablen persa con fluidez, que no hayan recibido ansiolíticos o antipsicóticos en los últimos seis meses, que no tengan problemas auditivos o cognitivos, sin antecedentes de estrés severo en los últimos tres meses, sin antecedentes de endoscopia o colonoscopia, y que obtuvieron al menos un 50% de la puntuación total en la lista de verificación de evaluación de necesidades del Modelo de Sistemas Neuman. C ^‡^: El estudio exploró los conceptos de variables del paciente (fisiológicas, psicológicas, socioculturales y de desarrollo), estresores intrapersonales, interpersonales y extrapersonales potenciales y reales, definición de metas, intervenciones y evaluación de enfermería, según el proceso de enfermería basado en el Modelo de Sistemas de Neuman. C ^§^: Atención médica domiciliaria.
Pereira F, Bieri M, Martins MM, Del Río Carral M, Verloo H ^(^ ^25^ ^)^ .	Italia/ 2022/ *Nursing Reports*	Cualitativo/ Estudio descriptivo	Identificar y categorizar los estresores experimentados y las estrategias de recuperación adoptadas por las personas mayores, sus cuidadores informales y los profesionales de la salud en el manejo de la medicación de las personas mayores después del alta hospitalaria.	P ^†^: Personas de 65 años o más hospitalizadas en los últimos 90 días a las que se les administra al menos cinco medicamentos diferentes por día. C ^‡^: El estudio exploró los conceptos de estresores extrapersonales y las estrategias de recuperación extrapersonales adoptadas por las personas mayores, sus cuidadores informales y los profesionales de la salud en el manejo de la medicación después del alta hospitalaria. C ^§^: Atención médica domiciliaria.

* PCC = Población, Concepto y Contexto;

† P = Población;

‡ C = Concepto;

§ C = Contexto

Entre los 14 estudios seleccionados, se encontró que la prevalencia de publicaciones fue mayor en 2021, y representa el 14,2% (n=2) de la muestra. Por otro lado, los otros 12 estudios se distribuyeron en diferentes años de publicación, cada año representa aproximadamente el 7,1% de la muestra y un solo estudio (n=1).

En cuanto al origen de los estudios, se destacó que el 50% (n=7) provenía de Estados Unidos (EE.UU.), mientras que el 14,2% (n=2) fueron realizados en Portugal y Brasil. El enfoque metodológico que predominó fue el cualitativo 71,4% (n=10), seguido del cuantitativo 21,4% (n=3) y del cuantitativo y cualitativo 7,1% (n=1).

## Discusión

Los estudios seleccionados se organizaron en dos categorías, considerando la similitud del contenido. La primera categoría aborda la implementación del Modelo de Sistemas de Neuman en contextos de atención hospitalaria, institucional y ambulatoria; y la segunda categoría implica el uso del modelo de sistemas de Neuman en los contextos comunitario y domiciliario.

### Categoría 1 - Implementación del Modelo de Sistemas de Neuman en ambientes hospitalarios, institucionales y ambulatorios

 Siete estudios analizaron la aplicabilidad del Modelo de Sistemas de Neuman en contextos hospitalarios, institucionales y ambulatorios ^(^ ^12^ ^-^ ^18^ ^)^ . Estas investigaciones abordaron condiciones específicas, tales como: atención al adulto mayor con cáncer asistido en el servicio de atención hospitalaria ^(^ ^12^ ^)^ ; atención al adulto mayor con artritis reumatoide atendido en un servicio de atención hospitalaria ^(^ ^13^ ^)^ ; prácticas de confort en residencias de adultos mayores de tamaño medio ^(^ ^14^ ^)^ ; fomentar la espiritualidad en residencias y unidades de cuidados de adultos mayores ^(^ ^25^ ^)^ ; prevención de lesiones por presión en una unidad de cuidados intensivos hospitalarios ^(^ ^16^ ^)^ ; y la identificación y el manejo de factores estresores durante la hospitalización, en clínica médica y clínica quirúrgica ^(^ ^17^ ^-^ ^18^ ^)^ . 

 El enfoque del Modelo de Sistemas demostró ser eficaz para abordar los factores estresores derivados de los efectos de la colostomía en la atención de una mujer mayor con cáncer, y abarcó diferentes dimensiones. A nivel intrapersonal, el cambio en los hábitos intestinales desencadenó estresores psicológicos, que generaron desafíos emocionales y psicológicos que afectaron la capacidad de adaptación de la persona mayor. En el ámbito interpersonal, la ausencia de apoyo social generó un impacto negativo adicional, que dificultó aún más el proceso de adaptación. Además, la hospitalización en sí representó un estresor en la dimensión extrapersonal, que afectó las interacciones sociales de la paciente. Las intervenciones de enfermería tuvieron como objetivo fortalecer las líneas de defensa de la mujer mayor, brindándole apoyo adaptativo y tratando de mantener su bienestar ideal ^(^ ^12^ ^)^ . 

 La necesidad de explorar en profundidad las creencias y los mecanismos para afrontar el cáncer, que se manifestó en el estudio de la mujer mayor con colostomía, se enfatiza en la efectividad de la implementación del Modelo de Sistemas de Neuman en pacientes con artritis reumatoide. La aplicación de este modelo a adultos mayores con artritis reumatoide ha demostrado ser efectiva al considerar de manera integral los impactos sociales, emocionales y físicos de esta condición crónica, e incorporar elementos como la espiritualidad y la capacidad de adaptación. Al analizar los efectos combinados de estos impactos en la percepción general de la salud de los adultos mayores, se destacó la importancia de la espiritualidad para afrontar las crisis agudas de la enfermedad. Además, el uso del modelo ha fomentado una colaboración más amplia entre pacientes y cuidadores, brindando alivio y soluciones a sus complejas necesidades de salud ^(^ ^13^ ^)^ . 

 También se observaron efectos beneficiosos del Modelo de Sistemas de Neuman, combinado con el confort para la autoestima, el bienestar, los procesos sociales, el estado de salud, la satisfacción con la vida, la autorrealización, la fe o creencia y la autorresponsabilidad de mujeres mayores que reciben atención en instituciones de cuidados a largo plazo ^(^ ^14^ ^)^ . 

 Los resultados del estudio sobre el confort mostraron que tuvo un impacto significativo en las percepciones de los adultos mayores, y promovió mejoras en el bienestar, la autoestima y otras variables evaluadas positivamente. Estos beneficios coinciden con los hallazgos del estudio que utilizó el Modelo de Sistemas para prevenir lesiones por presión en pacientes hospitalizados, y destacan la efectividad de enfoques que consideran no solo los factores físicos, sino también aspectos emocionales, sociales y ambientales para mejorar la salud y el bienestar de los pacientes. La aplicación combinada del Modelo de Sistemas con la escala de evaluación del riesgo de lesión por presión demostró la efectividad que tiene en la prevención primaria, y le permitió al equipo de enfermería adoptar medidas preventivas específicas para reducir potenciales estresores y fortalecer las defensas de los pacientes contra el estrés ^(^ ^16^ ^)^ . 

 En otra investigación se destacó la relevancia de la educación para la salud para brindar una atención holística, que considera la espiritualidad de los adultos mayores según el Modelo de Neuman. El estudio realizado en residencias y unidades de atención de adultos mayores reveló que tiene efectos positivos sobre la salud física y mental, y que fortalece las relaciones interpersonales y la capacidad de afrontar desafíos. Se observó que a medida que la salud de los adultos mayores se deteriora, aumenta la dependencia espiritual, lo que indica que desean recibir atención espiritual por parte de los profesionales de la salud ^(^ ^15^ ^)^ . 

 El uso del Modelo de Sistemas de Neuman para identificar y gestionar estresores durante la hospitalización de adultos mayores también demostró que ciertos estresores afectan varias áreas clínicas y personales, tales como: edad avanzada, capacidad física reducida, conocimientos limitados y emociones negativas, además afectan las relaciones familiares, la privacidad, la autonomía y el ambiente hospitalario ^(^ ^17^ ^)^ . Dichos hallazgos han servido de base para implementar intervenciones de enfermería centradas en el síndrome del adulto mayor frágil, los trastornos del sueño, la ansiedad, el miedo y sufrimiento espiritual, con el fin de reducir el impacto de esos factores estresores y mejorar la experiencia de los adultos mayores durante su estancia en el hospital ^(^ ^18^ ^)^ . 

Los hallazgos de estos estudios destacan la eficacia que tienen las intervenciones terapéuticas en ambientes hospitalarios, institucionales y ambulatorios para mejorar la autoestima y el bienestar de los pacientes. La aplicación del Modelo de Sistemas de Neuman reveló un impacto significativo en la calidad de vida de los adultos mayores, y abarcó dimensiones físicas, psicológicas, sociales y espirituales. El enfoque de la espiritualidad demuestra que es un elemento fundamental en las intervenciones de salud, que afecta la percepción del propósito de vida y la capacidad para afrontar los factores estresores. Cabe destacar la importancia que tiene la interacción entre adultos mayores, familiares y profesionales de la salud para promover vínculos afectivos y de confianza, que fortalezcan las líneas de defensa y resistencia, y den como resultado intervenciones efectivas que tengan un impacto positivo en sus vidas.

### Categoría 2 - Uso del Modelo de Sistemas de Neuman en el ambiente comunitario y domiciliario

 Esta categoría abarca siete estudios ^(^ ^19^ ^-^ ^25^ ^)^ , que estudiaron la aplicación del Modelo de Sistemas de Neuman en el ambiente comunitario y el domiciliario, que se centran en la atención para promover la salud de los adultos mayores a través de: atención domiciliaria a los adultos mayores después de un infarto agudo de miocardio ^(^ ^19^ ^)^ ; apoyo domiciliario post alta hospitalaria tras un episodio de accidente cerebrovascular (ACV) ^(^ ^20^ ^)^ ; atención a adultos mayores con Parkinson atendidos por el servicio comunitario de salud ^(^ ^21^ ^)^ ; actividades de educación para la salud realizadas por el servicio comunitario de salud ^(^ ^22^ ^)^ ; Intervención de Práctica Colaborativa Interprofesional (IPCP) para adultos mayores que viven en la comunidad ^(^ ^23^ ^)^ ; reducción de la ansiedad en adultos mayores que esperan una colonoscopia en su domicilio ^(^ ^24^ ^)^ ; y manejo de medicamentos después del alta hospitalaria ^(^ ^25^ ^)^ . 

El Modelo de Sistemas de Neuman demostró que se puede aplicar en el ambiente comunitario y el domiciliario, e incluye la familia, la comunidad y el hogar. Permitió realizar una evaluación integral de las necesidades y recursos de las personas y familias, posibilitó la implementación de intervenciones preventivas, curativas y de rehabilitación. Además, el modelo valoró la participación activa de los individuos y las familias en la atención de la salud, fortaleciendo el vínculo entre los profesionales de la salud y la comunidad.

 Este modelo ha demostrado ser eficaz para la recuperación domiciliaria de adultos mayores después de sufrir un infarto agudo de miocardio, al promover su bienestar según sus valores personales, establecer metas progresivas y utilizar adecuadamente los recursos disponibles ^(^ ^18^ ^)^ . 

 Por lo tanto, la aplicación del Modelo de Sistemas de Neuman en la prevención terciaria demostró mejoras significativas en el restablecimiento del equilibrio del sistema paciente después de un infarto agudo de miocardio, y arrojó resultados similares en un estudio sobre acciones preventivas dirigidas a la recuperación de adultos mayores después del alta hospitalaria luego de un ACV. El estudio permitió identificar factores estresores individuales y familiares, como: privación de sueño, ansiedad, dificultades de comunicación y falta de apoyo externo, con un plan de cuidados enfocado en promover la autonomía, optimizar los patrones de sueño e implementar ajustes ambientales para facilitar la recuperación. Estas estrategias incluyeron intervenciones motivacionales, educativas y conductuales tanto en el contexto individual como familiar, y destacaron la adaptabilidad y efectividad del modelo en diferentes escenarios clínicos ^(^ ^19^ ^)^ . 

 La aplicación del Modelo de Sistemas de Neuman les permitió a los enfermeros establecer objetivos de atención efectivos mediante intervenciones de prevención primaria, secundaria y terciaria; estos coincidían con las necesidades de salud a corto, mediano y largo plazo de una pareja de adultos mayores, en la que uno de los miembros fue diagnosticado con la enfermedad de Parkinson. Estos objetivos abarcaron diferentes aspectos, como el apoyo emocional, el manejo de la incontinencia nocturna y el afrontamiento de los miedos de los adultos mayores ante la pérdida de independencia. Por medio de una comunicación eficiente entre los profesionales de la salud involucrados, se pudo garantizar la continuidad de la atención y brindar el apoyo necesario, lo que provocó una mejora en la calidad del sueño del adulto mayor y una reducción del aislamiento social de la esposa, quien desempeñaba el papel de cuidadora ^(^ ^20^ ^)^ . 

 Otro punto crucial fue el uso del Modelo de Sistemas de Neuman como una sólida herramienta de atención longitudinal a través del servicio de salud comunitario. Las acciones de educación para la salud han proporcionado mejoras significativas en el bienestar de los adultos mayores, dotándolos de conocimientos sobre medidas preventivas, fomentando cambios en el estilo de vida y promoviendo el desarrollo de redes de apoyo. Las mismas tuvieron un impacto particularmente notorio en los adultos mayores de bajos ingresos, fomentaron la promoción de la salud y una mejor calidad de vida ^(^ ^21^ ^)^ . 

 Por lo tanto, se comprueba que la implementación del Modelo de Sistemas de Neuman en la atención comunitaria juega un papel crucial en el bienestar de los adultos mayores. Un estudio realizado en el servicio comunitario de salud identificó la contribución de los factores estresores que impactan sobre la calidad de vida de los adultos mayores, seguida del desarrollo de estrategias de prevención primaria, secundaria y terciaria, para fortalecer las líneas de defensa y resistencia de los adultos mayores y promover el bienestar de los mismos ^(^ ^23^ ^)^ . 

 También se obtuvieron resultados positivos sobre la reducción de la ansiedad en adultos mayores que esperan una colonoscopia en casa, a través de intervenciones específicas dirigidas a cada estresor identificado, como preparación intestinal, problemas de salud, trastornos del sueño, miedo al diagnóstico, falta de conocimiento y aislamiento social ^(^ ^24^ ^)^ . 

 En cuanto a la gestión de la medicación en el domicilio después del alta hospitalaria de los adultos mayores, la identificación de factores estresores relacionados con este proceso reveló que había problemas de comunicación, colaboración y coordinación de la atención entre los adultos mayores, los cuidadores informales y los profesionales de la salud. La falta de participación de los adultos mayores en las decisiones sobre la medicación generó preocupación, lo que provocó dificultades para comprender y cumplir el tratamiento. Por lo tanto, cabe destacar que es importante que se adopte un enfoque centrado en el paciente, interprofesional y colaborativo para mejorar la seguridad en la gestión de medicamentos ^(^ ^25^ ^)^ . 

Los estudios presentados confirman el papel crucial de los enfermeros en la promoción de la salud de los adultos mayores, que fortalecen sus redes de apoyo e implementan estrategias de prevención primaria, secundaria y terciaria en contextos comunitarios y domiciliarios, con el objetivo de mantener el bienestar y el equilibrio del sistema paciente.

En lo que respecta a las lagunas de conocimiento identificadas, se observó que faltaban propuestas participativas, horizontales e intergeneracionales para afrontar eficazmente los factores estresores durante toda la vida considerando el envejecimiento saludable. Además, el estudio destacó la falta de investigaciones que aborden el papel del enfermero como miembro de equipos multidisciplinarios, que se enfocan en identificar factores estresores e implementar intervenciones adecuadas en diferentes contextos de atención, con el objetivo de promover un envejecimiento saludable y un afrontamiento eficaz de los factores estresores durante toda la vida.

Este estudio tuvo algunas limitaciones. No se pudo acceder a tres publicaciones completas, lo que restringió la posibilidad de realizar un análisis profundo de estos documentos. Además, es posible que la estrategia de búsqueda empleada no haya incluido todos los términos relevantes, especialmente cuando se considera el contexto “servicios de salud”, dado que este término presenta una amplia variación terminológica en diferentes naciones. Extender la revisión a otras bases de datos relevantes también podría haber ampliado el alcance del estudio, y permitir que se incorporaran investigaciones adicionales sobre el tema propuesto.

## Conclusión

Se comprobó que la integración del enfoque sistémico de Neuman en la práctica de enfermería gerontológica, en diferentes ambientes asistenciales como el hospitalario, institucional, ambulatorio, comunitario y domiciliario, era efectiva y adaptable, dado que considera a los adultos mayores como individuos integrales y multidimensionales.

Este enfoque satisface las diferentes necesidades de los adultos mayores al considerar las cinco variables del paciente: fisiológicas; psicológicas; espirituales; socioculturales; y de desarrollo, implementa acciones de cuidado para fortalecer las líneas de defensa y resistencia del paciente, además reconoce los estresores potenciales y reales del paciente (intrapersonales, interpersonales y extrapersonales), y utiliza estrategias de intervención primarias, secundarias y terciarias para mejorar su bienestar y calidad de vida.

El despliegue de esta investigación implica el desarrollo de estudios dirigidos a promover el envejecimiento saludable, considerando los elementos conceptuales de la teoría y las complejidades inherentes a la vejez, con el objetivo de mejorar las prácticas de enfermería en beneficio del bienestar de la población que envejece.

# Translation: Aplicabilidade do Modelo de Sistemas de Neuman à prática de enfermagem gerontológica: revisão de escopo

**DOI:** 10.1590/1518-8345.6977.4225

**Authors:** Samara Gonçalves de Oliveira, Célia Pereira Caldas, Esther Mourão Nicoli, Frances Valéria Costa e Silva, Rosane Barreto Cardoso, Fernanda Maria do Vale Martins Lopes

**Affiliations:** 1 Universidade do Estado do Rio de Janeiro, Faculdade de Enfermagem, Rio de Janeiro, RJ, Brasil.; 2 Bolsista da Fundação Carlos Chagas Filho de Amparo à Pesquisa do Estado do Rio de Janeiro (FAPERJ), Brasil.; 3 Universidade Federal do Rio de Janeiro, Faculdade de Enfermagem, Rio de Janeiro, RJ, Brasil.

**Keywords:** Idoso, Serviços de Saúde, Enfermagem, Teoria de Enfermagem, Cuidados de Enfermagem, Teoria de Sistemas

## Abstract

**Objetivo:**

mapear as produções científicas sobre a aplicação do Modelo de Sistemas de Neuman à prática de enfermagem voltada para a atenção à saúde de idosos.

**Método:**

revisão de escopo, baseada na metodologia proposta pelo *Joanna Briggs Institute* . Foram consultadas sete bases de dados eletrônicas. Quanto aos critérios de elegibilidade, consideraram-se: População - idosos; Conceito - aplicação do Modelo de Sistemas de Neuman na prática de enfermagem; e Contexto da pesquisa - serviços de saúde.

**Resultados:**

14 estudos compuseram a amostra. Os dados foram analisados e sumarizados em duas categorias: implementação do Modelo de Sistemas de Neuman em ambientes hospitalares, institucionais e ambulatoriais e utilização do Modelo de Sistemas de Neuman em ambientes comunitário e domiciliar.

**Conclusão:**

A aplicação da abordagem de sistemas de Neuman à prática de enfermagem gerontológica, em diversos cenários de cuidado revelou-se promissora, ao considerar os idosos como indivíduos abrangentes e de múltiplas dimensões. Essa perspectiva demonstrou adaptabilidade e eficácia para atender às diversas necessidades dos idosos, resultando em uma melhoria em sua qualidade de vida na velhice.

## Destaques

**(1)** É possível integrar a abordagem sistêmica na prática de enfermagem gerontológica.

**(2)** O Modelo de Sistemas abrange de forma integrativa as necessidades dos idosos.

**(3)** Ações para fortalecer as linhas de defesa e resistência do idoso são importantes.

**(4)** O Modelo favorece a identificação de fatores estressores potenciais e reais do idoso.

**(5)** Requer-se estudos com abordagem sistêmica para promover o envelhecimento saudável.

## Introdução

 A atuação do enfermeiro possibilita o oferecimento de suporte adequado ao indivíduo, por meio da identificação minuciosa de suas necessidades de cuidado, assim como no desenvolvimento de ações que promovam a saúde em distintos contextos de atenção e em todos os ciclos da vida. Portanto, sua ação na assistência aos idosos pode contribuir significativamente para melhorar a saúde e o bem-estar dessa população, considerando o envelhecimento populacional, fenômeno mundial que adquire crescente visibilidade no âmbito da saúde nas últimas décadas ^(^ ^1^ ^-^ ^2^ ^)^ . 

 O Modelo de Sistemas de Neuman é uma abordagem teórica que pode ser utilizada para compreender o comportamento dos idosos em relação ao processo de saúde e doença ao longo da vida, considerando o envelhecimento. Essa abordagem se baseia em um sistema holístico que concentra sua atenção na interação do indivíduo com seu ambiente singular, sendo uma ferramenta valiosa para os enfermeiros que buscam compreender as complexidades do envelhecimento ^(^ ^3^ ^-^ ^4^ ^)^ . 

 Na visão sistêmica deste modelo, o homem e seu ambiente são considerados um sistema aberto, representado por círculos concêntricos que incluem o núcleo, as linhas de resistência e as linhas de defesa. Intervenções de enfermagem visam evitar a penetração de estressores no núcleo e auxiliar a restauração das linhas de resistência e defesa ^(^ ^3^ ^)^ . 

 O modelo postula que a saúde é um estado dinâmico de equilíbrio entre o indivíduo e seu ambiente e que quando esse equilíbrio é perdido, há ruptura das barreiras de defesa, que se expressa como adoecimento. A meta do cuidado de enfermagem é auxiliar o indivíduo a atingir e manter o equilíbrio adequado por meio de intervenções apropriadas. O indivíduo é percebido como um sistema aberto interativo que interage com estressores ambientais internos ou externos. Devido à historicidade e dinamicidade de cada indivíduo, que está em constante mudança devido à interação ambiental recíproca, a falta de atendimento de suas necessidades de saúde reduz a condição de bem-estar subjetivo ^(^ ^5^ ^)^ . 

 A amplitude conceitual, a flexibilidade e propriedades sistêmicas do Modelo de Sistemas de Neuman fornecem marcadores essenciais para uma visão integrada de liderança e erudição, em conformidade com a filosofia de enfermagem, visando aprimorar as atividades profissionais. Sendo uma concepção teórica holística, suas principais características são a confiabilidade, a viabilidade, a flexibilidade e a abrangência. Tais características são fundamentais para expansão dos papéis e responsabilidades profissionais da enfermagem no século XXI ^(^ ^6^ ^)^ . 

Nesta perspectiva, destaca-se a pertinência desta revisão de escopo que visa refletir sobre a aplicação do Modelo de Sistemas de Neuman no contexto da enfermagem gerontológica. Este estudo se justifica pela sua possibilidade de contribuir para reflexões sobre as implicações do cuidado de enfermagem prestado aos idosos com base no Modelo de Sistemas de Neuman.

O objetivo desta pesquisa é mapear as produções científicas sobre a aplicação do Modelo de Sistemas de Neuman na prática de enfermagem voltada para a atenção à saúde de idosos.

## Método

 Trata-se de uma revisão de escopo guiada pelas recomendações da *Joanna Briggs Institute Reviewer’s Manual* (JBI), executada em nove etapas: 1) identificação da questão de pesquisa; 2) identificação de estudos relevantes; 3) seleção de estudos; 4) mapeamento de dados; 5) compilação dos resultados; 6) extração das evidências; 7) análise das evidências; 8) apresentação dos resultados; e 9) resumo das evidências, conclusões e implicações dos achados ^(^ ^7^ ^)^ . 

 Seguiram-se as recomendações das diretrizes contidas no *checklist* do *Preferred Reporting Items for Systematic reviews and Meta-Analyses extension for Scoping Reviews* (PRISMA-ScR) ^(^ ^8^ ^)^ . 

 Para a construção da estratégia de busca e questão de pesquisa foi utilizado o mnemônico “PCC” (população, conceito e contexto), recomendado pelo JBI como guia para construir, além do título e dos critérios de inclusão, a questão de pesquisa ^(^ ^7^ ^,^ ^9^ ^)^ . 

A questão de pesquisa foi formulada considerando: População – idosos; Conceito – aplicação do Modelo de Sistemas de Neuman à prática de enfermagem; e Contexto da pesquisa – serviços de saúde, sendo assim estruturada: Como o Modelo de Sistemas de Neuman tem sido aplicado à prática de enfermagem voltada para a atenção à saúde de idosos?

 A estratégia de busca foi concebida contando com a colaboração de duas bibliotecárias, para identificar estudos relevantes para esta revisão. O objetivo foi analisar o vocabulário controlado e seus sinônimos, adaptando a sintaxe de busca a cada fonte de dados. Inicialmente, realizou-se uma pesquisa preliminar em 25 de abril de 2023 nas bases de dados *Medical Literature Analysis and Retrieval System Online* (MEDLINE) via PubMed e na *Cumulative Index to Nursing & Allied Health Literature* (CINAHL). Essa etapa visou garantir consistência no processo de seleção dos estudos, revelando um número limitado de evidências sobre o tema proposto e a ausência de revisões relacionadas. 

### Protocolo e registro

 Elaborou-se o protocolo para a revisão de escopo, registrado no *Open Science Framework* (OSF), com o identificador DOI: https://doi.org/10.17605/OSF.IO/9DCE6 . 

### Critérios de elegibilidade

 Como critérios de elegibilidade, foram consideradas: obras científicas que discorressem sobre a utilização da perspectiva teórica do Modelo de Sistemas de Neuman na atenção à saúde da pessoa idosa no âmbito dos serviços de saúde; e estudos que utilizassem como amostra a população idosa, definida como “pessoas com idade igual ou superior a 60 anos”, conforme o Estatuto do Idoso ^(^ ^10^ ^)^ . Produções científicas com amostragem heterogênea, ou seja, que incluíam outras faixas etárias além dos idosos, foram excluídas. 

Quanto aos tipos de fontes, foram incluídos estudos de abordagem qualitativa, quantitativa e de método misto. A incorporação de estudos quantitativos, qualitativos e de método misto visou capturar uma gama diversificada de perspectivas e evidências, proporcionando uma análise holística do tema. Teve-se em mente, com a inclusão destas abordagens metodológicas, proporcionar uma compreensão mais robusta e contextualizada do conhecimento disponível acerca da aplicabilidade do Modelo de Sistemas de Neuman, na prática de enfermagem gerontológica.

Foram excluídos os estudos não relacionados ao tema, sem metodologia definida, incompletos, de revisão, editoriais, capítulos de livro, documentos governamentais (leis, decretos, portarias), resenhas, glossários de termos científicos, anais, textos e artigos de opinião, cartas, livros, teses, dissertações e resumos de eventos.

### Fontes de informação

 A pesquisa das produções científicas foi conduzida nas plataformas de literatura acadêmica *ScienceDirect* (Elsevier) e *Web of Science* , além das bases de dados MEDLINE via PubMed, CINAHL, Embase, Literatura Latino-Americana e do Caribe em Ciências da Saúde (LILACS), e Scopus, sem restrição de data de publicação. 

### Pesquisa

A pesquisa dos estudos foi conduzida em 29 de maio, enquanto a seleção ocorreu nos meses de maio e junho de 2023.

### Seleção de fontes de evidência

 As etapas de seleção dos estudos nas bases de dados foram: 1. Exportação dos resultados obtidos para um gerenciador de referências; 2. Eliminação das duplicatas; 3. Transferência dos arquivos para o *software Rayyan Qatar Computing Research Institute* (QCRI), que permite o cegamento na colaboração entre os revisores ^(^ ^11^ ^)^ ; 4. Seleção dos estudos conforme a análise do título e resumo de maneira independente e duplo cega segundo os critérios de análise pré-estabelecidos para inclusão ou exclusão dos estudos; 5. Resolução de discordâncias no processo de seleção por um terceiro revisor; 6. Leitura dos textos completos dos artigos; 7. Seleção final dos textos incluídos na revisão. 

### Processo de mapeamento de dados

 Para mapeamento dos dados, utilizou-se a estratégia de busca com os operadores booleanos ‘AND’ e ‘OR’, incorporando vocabulários controlados e seus sinônimos. Não foram aplicados filtros relacionados ao idioma e ao limite temporal, para garantir a inclusão de todas as fontes de literatura nacional e internacional. A estratégia de busca traçada para as fontes de dados é ilustrada a seguir na Figura 1 . 

**Figura 1:** - Estratégia de busca desenvolvida, adaptada a cada fonte de dados. Rio de Janeiro, RJ, Brasil, 2023

**Fontes de dados**	**Estratégia de Busca**	**Quantitativo de produções científicas**
**MEDLINE via PubMed**	(aged[MeSH] OR aged OR “aged patient” OR “aged subject” OR elderly OR “elderly population” OR “elderly patient” OR “elderly subject” OR senior OR “senior citizens” OR old OR “old-aged” OR “old people” OR “old person” OR “older people” OR “aged, 80 and over”[MeSH] OR “aged, 80 and over” OR “Oldest Old” OR “very elderly” OR “very old” OR “very elderly” OR eldest OR elder OR “middle aged”[MeSH] OR “middle aged”) AND (“neuman systems model” OR “neuman model” OR “neuman`s model” OR “neuman`s systems model”)	49
**CINAHL**	( (MH “Aged”) OR “aged” OR “elderly” OR ““elderly population”” OR “senior” OR ““senior citizens”” OR “old” OR ““old-aged”” OR ““old people”” OR ““older people”” OR “elder” OR “eldest” OR (MH “Aged, 80 and Over”) OR ““Aged, 80 and over”” OR ““Oldest Old”” OR (MH “Middle Age”) OR ““Middle Aged”” ) AND ( (MH “Neuman Systems Model”) OR “neuman systems model” OR ““neuman model”” OR ““neuman`s model”” OR ““neuman`s systems model”” )	40
**Embase**	(‘aged’/de OR ‘aged patient’ OR ‘aged people’ OR ‘aged person’ OR ‘aged subject’ OR ‘elderly’ OR ‘elderly patient’ OR ‘elderly people’ OR ‘elderly person’ OR ‘elderly subject’ OR ‘senior citizen’ OR ‘senior’ OR ‘senior citizens’ OR ‘aged’ OR ‘very elderly’/de OR ‘aged, 80 and over’ OR ‘very old’ OR ‘very elderly’ OR ‘middle aged’/de OR ‘middle age’ OR ‘middle aged’) AND (‘neuman systems model’/de OR ‘neuman model’ OR ‘neuman`s model’ OR ‘neuman`s systems model’ OR ‘neuman systems model’)	36
***Web of Science***	TS=((aged OR “aged patient” OR “aged subject” OR elderly OR “elderly population” OR “elderly patient” OR “elderly subject” OR senior OR “senior citizens” OR old OR “old-aged” OR “old people” OR “old person” OR “older people” OR “aged, 80 and over” OR “Oldest Old” OR “very elderly” OR “very old” OR “very elderly” OR eldest OR elder OR “middle aged” OR “middle age”) AND (“neuman systems model” OR “neuman model” OR “neuman`s model” OR “neuman`s systems model”))	20
**Scopus**	TITLE-ABS-KEY ( ( aged OR “aged patient” OR “aged subject” OR elderly OR “elderly population” OR “elderly patient” OR “elderly subject” OR senior OR “senior citizens” OR old OR “old-aged” OR “old people” OR “old person” OR “older people” OR “aged, 80 and over” OR “oldest old” OR “very elderly” OR “very old” OR “very elderly” OR eldest OR elder OR “middle aged” OR “Middle Age” ) AND ( “neuman systems model” OR “neuman model” OR “neuman`s model” OR “neuman`s systems model” ) )	59
***Science Direct***	(aged OR elderly OR “middle aged” OR “aged, 80 and over”) AND (“neuman systems model” OR “neuman model” OR “neuman`s model” OR “neuman`s systems model” OR “neuman systems model”)	112
**LILACS**	((mh:(“aged”) OR aged OR elderly OR “elderly population” OR senior OR “senior citizens” OR old OR “old-aged” OR “old people” OR “old person” OR “older people” OR elder OR eldest OR mh:(“aged, 80 and over”) OR “aged, 80 and over” OR “oldest old” OR mh:(“middle aged”) OR “middle aged”)) AND ((“neuman systems model” OR “neuman model” OR “neuman`s model” OR “neuman`s systems model”))	6

### Itens de dados

 As informações dos textos incluídos foram organizadas e armazenadas em uma planilha no programa Excel ^®^ para facilitar a extração das evidências. Foram considerados os seguintes itens de dados: título; tempo (ano que foi publicado); autores; fonte de dados; local (país de origem); propósitos (objetivos); principais resultados; população de estudo (requisitos para a participação no estudo); desenho do estudo (método); fatores estressores; estratégias eficazes de enfrentamento de estressores; população (pessoas com idade maior ou igual a 60 anos); aplicação do Modelo de Sistemas, à prática de enfermagem; possibilidades de cuidado de enfermagem; e contexto (serviço de saúde em que foi realizado o cuidado de enfermagem alicerçado no Modelo de Sistemas). 

### Tratamento e análise dos dados

Com o propósito de identificar os componentes essenciais de cada estudo, foi adotada uma estrutura analítica descritiva para examinar criticamente o conteúdo de cada investigação selecionada.

O sumário dos resultados foi realizado ao identificar as principais temáticas abordadas nos estudos, permitindo a análise da recorrência, convergência e divergência dos conteúdos. Essa abordagem possibilitou a compreensão da aplicabilidade do Modelo de Sistemas de Neuman, à prática de enfermagem gerontológica, resultando no delineamento de categorias para discussão dos achados.

### Aspectos éticos

Por tratar-se de uma pesquisa com dados secundários, de domínio público e disponíveis na literatura, não houve a necessidade de apreciação ética. Contudo, cabe destacar que foram respeitados os direitos autorais com correta citação e referenciamento dos estudos.

## Resultados

 Foram identificadas, inicialmente, 322 produções científicas relevantes. Obteve-se uma amostra final de 14 estudos. A Figura 2 ilustra o caminho percorrido pelo processo de seleção dos estudos, desde a identificação inicial até a constituição da amostra final para análise e discussão. 

 Com base em seu conteúdo, os estudos foram dispostos em duas categorias: implementação do Modelo de Sistemas de Neuman em ambientes hospitalares, institucionais e ambulatoriais; e utilização do Modelo de Sistemas de Neuman em ambientes comunitário e domiciliar. A Figura 3 apresenta a caracterização dos estudos selecionados na revisão de escopo, segundo país, ano de publicação, abordagem metodológica e periódico científico. 

**Figura 2:** - Fluxograma de seleção dos estudos segundo recomendações *Preferred Reporting Items for Systematic Review and Meta-Analysis extension for Scoping Reviews* . Rio de Janeiro, RJ, Brasil, 2023 ^(^ ^8^ ^)^

*n=número de registros

**Figura 3:** - Produções científicas selecionadas quanto a natureza das fontes de publicação. Rio de Janeiro, RJ, Brasil, 2023

**Categoria 1- Implementação do Modelo de Sistemas de Neuman em ambientes hospitalares, institucionais e ambulatoriais**				
**Referência**	**País/Ano de Publicação/Fonte de dados**	**Abordagem metodológica/Tipo de estudo**	**Objetivos**	**PCC** ^*^
Weinberger SL ^(^ ^12^ ^)^ .	Estados Unidos/ 1991/ *Rehabilitation Nursing*	Qualitativa/ Relato de caso	Descrever uma análise usando o *Neuman Systems Model* para determinar os efeitos de uma colostomia em um cliente de 81 anos com câncer.	P ^†^: Pessoa de 81 anos diagnosticado com câncer de intestino e em uso de bolsa de colostomia. C ^‡^: O estudo explorou os conceitos de estressores intrapessoais, interpessoais, extrapessoais, as variáveis do cliente psicológicas, socioculturais e fisiológicas, bem como as linhas de defesa e ações de prevenção secundária. C ^§^: Serviço de saúde hospitalar.
Potter ML, Zauszniewski JA ^(^ ^13^ ^)^ .	Estados Unidos/ 2000/ *Journal of Holistic Nursing*	Quantitativa/Estudo correlacional e transversal	Examinar variáveis que refletem reações ao estresse, linhas de defesa e resistência e o núcleo básico dos seres humanos no contexto do Modelo de Sistemas de Neuman em uma amostra de idosos	P ^†^: Pessoas com idade maior ou igual a 60 anos com diagnóstico de artrite reumatoide. C ^‡^: O estudo explorou os conceitos de variáveis do sistema cliente (fisiológicas, psicológicas, socioculturais, espirituais e de desenvolvimento), linhas de defesa e resistência e o núcleo básico do cliente. C ^§^: Serviço de saúde hospitalar.
Butts JB ^(^ ^14^ ^)^ .	Estados Unidos/ 2001/ *Geriatric Nursing*	Quantitativa/ Estudo experimental	Examinar se o toque de conforto melhorou as percepções de autoestima, bem-estar e processos sociais, estado de saúde, satisfação com a vida e autorrealização e fé ou crença e autorresponsabilidade de idosas institucionalizadas.	P ^†^: Mulher com idade maior ou igual a 65 anos. C ^‡^: O estudo explorou os conceitos de variáveis do sistema cliente (fisiológicas, psicológicas, socioculturais, espirituais e de desenvolvimento) para examinar se o toque de conforto melhorou as percepções de autoestima, bem-estar e processos sociais, estado de saúde, satisfação com a vida e autorrealização e fé ou crença e autorresponsabilidade. C ^§^: Duas casas de repouso de médio porte.
Lowry LW ^(^ ^15^ ^)^ .	Estados Unidos/ 2012/ *Nursing Science Quarterly*	Qualitativa/ Descritivo e interpretativo	Explorar o significado da espiritualidade conforme descrito por adultos idosos em vários estados de saúde, descrever a relação entre espiritualidade e saúde e explicar as expectativas dos clientes para os profissionais de saúde relacionadas à espiritualidade.	P ^†^: Pessoa com idade maior ou igual a 60 anos. C ^‡^: o estudo utilizou o conceito da variável espiritualidade do Modelo de Sistemas de Neuman. C ^§^: Casas de repouso e unidades de cuidado de enfermagem.
Sousa JERB, Silva GRF, Luz MHBA, Pereira MLL ^(^ ^16^ ^)^ .	Espanha/ 2015/ *Index de Enfermería*	Qualitativa/ Relato de caso	Mostrar como a utilização da Teoria de Neuman associada à prevenção de lesão por pressão utilizando a Escala de Braden como padrão clínico, é eficaz na redução da incidência de lesão por pressão na unidade de terapia intensiva.	P ^†^: Mulher de 94 anos com insuficiência respiratória aguda, pneumonia e diabetes descompensada. C ^‡^: O estudo investigou as variáveis do cliente (fisiológicas, psicológicas, socioculturais e de desenvolvimento). Além disso, examinou a natureza e intensidade de fatores estressores intrapessoais, interpessoais e extrapessoais, considerando sua influência na probabilidade do surgimento e reação a esses estressores. Analisou também a interação do cliente com os estressores ambientais, estratégias de cuidado para manter a estabilidade do sistema cliente e alcançar o mais alto nível possível de bem-estar geral do cliente. C ^§^: Serviço de atenção hospitalar (unidade de terapia intensiva).
Rosa PH, Beuter M, Benetti ERR, Bruinsma JL, Venturini L, Backes C ^(^ ^17^ ^)^ .	Brasil/ 2018/ Escola Anna Nery Revista de Enfermagem	Qualitativa/ Estudo descritivo	Descrever os estressores vivenciados por idosos hospitalizados na perspectiva do Modelo de Sistemas de Neuman	P ^†^: Pessoa com idade maior ou igual a 60 anos hospitalizadas há mais de cinco dias. C ^‡^: O estudo explorou os conceitos de estressores intrapessoais, interpessoais e extrapessoais do sistema cliente. C ^§^: Serviço de atenção hospitalar (clínica médica).
Benetti ERR, Beuter M, Rosa PH, Backes C, Jacobi CS, Oliveira FF ^(^ ^18^ ^)^ .	Brasil/ 2021/ Revista de Enfermagem da Universidade Federal de Santa Maria	Qualitativa/ Pesquisa Convergente Assistencial	Caracterizar as pessoas idosas hospitalizadas quanto às dimensões propostas pelo Modelo de Sistemas de Neuman.	P ^†^: Pessoas com idade maior ou igual a 60 anos hospitalizadas. C ^‡^: O estudo explorou o Processo de Enfermagem embasado neste modelo teórico, evidenciando os conceitos de: variáveis do cliente (fisiológicas, psicológicas, socioculturais e de desenvolvimento), fatores estressores, estratégias de enfrentamento relacionados aos fatores estressores e ações de cuidado para promover a estabilidade do sistema cliente. C ^§^: Serviço de atenção hospitalar (clínica Médica I; Clínica Médica II e Clínica Cirúrgica).
**Categoria 2 - Utilização do Modelo de Sistemas de Neuman em ambientes comunitário e domiciliar**				
**Referência**	**País/Ano de Publicação**	**Abordagem metodológica**	**Periódico científico**	PCC ^*^
Ross MM, Bourbonnais FF ^(^ ^19^ ^)^ .	Reino Unido/ 1985/ *Journal of Advanced Nursing*	Qualitativa/ Relato de caso.	Descrever o uso do Modelo de Sistemas Betty Neuman, na prática, usando uma abordagem de relato de caso.	P ^†^: Pessoa com 66 anos de idade em processo de recuperação domiciliar após hospitalização e tratamento de um infarto do miocárdio. C ^‡^: O estudo explorou os conceitos de estressores intrapessoais, interpessoais e extrapessoais, linhas de defesa, linhas de resistência do sistema cliente e estratégias de intervenção primária, secundária e terciária para promover a estabilidade do sistema cliente. C ^§^: Serviço de atenção domiciliar.
Ross MM, Helmer H ^(^ ^20^ ^)^ .	Estados Unidos/ 1988/ *Public Health Nursing*	Qualitativa/ Relato de caso.	Identificar as semelhanças e diferenças entre cuidar de um indivíduo como cliente e cuidar de uma família como cliente quando se usa o modelo de sistemas de Betty Neuman como um guia para a prática.	P ^†^: Um casal de idosos (a mulher de 63 anos e o homem de 66 anos). C ^‡^: O estudo explorou os conceitos de estressores intrapessoais, interpessoais e extrapessoais, linhas de defesa, linhas de resistência do sistema cliente e estratégias de intervenção primária, secundária e terciária para promover a estabilidade do sistema cliente. C ^§^: Serviço de atenção domiciliar.
Millard J ^(^ ^21^ ^)^ .	Reino Unido/ 1992/ *British Journal of Nursing*	Qualitativa/ Relato de caso	Analisar o papel dos visitadores de saúde no envolvimento com a população idosa, examinando como os instrumentos de avaliação podem influenciar a percepção do cliente quanto à obtenção de informações e o cuidado/apoio oferecido.	P ^†^: Pessoa com idade maior ou igual a 60 anos com Parkinson. C ^‡^: O estudo explorou os conceitos de estressores intrapessoais, interpessoais e extrapessoais, delineando objetivos de cuidado em curto, médio e longo prazos com base nas necessidades de saúde do cliente por meio de intervenções de prevenção primária, secundária e terciária. C ^§^: Serviço de saúde comunitário.
Imamura E ^(^ ^22^ ^)^ .	Estados Unidos/ 2002/ *International Journal of Nursing Practice*	Qualitativa/ Estudo de Intervenção	Promover a compreensão dos idosos sobre as doenças crônicas; informá-los sobre nutrição adequada e seu impacto sobre sua saúde; realizar exercícios específicos para idosos; e facilitar a construção de relacionamentos de apoio entre adultos idosos.	P ^†^: Pessoas com idade maior ou igual a 65 anos. C ^‡^: um questionário multidimensional, baseado nos conceitos de variáveis do cliente (fisiológicas, psicológicas, socioculturais, espirituais e de desenvolvimento), foi desenvolvido para avaliar as experiências individuais e comportamentais e explorar as condições de saúde dos participantes. C ^§^: Serviço de saúde comunitário.
Montano AR, Shellman J, Malcolm M, McDonald D, Rees C, Fortinsky R, Reagan L ^(^ ^23^ ^)^ .	Estados Unidos/ 2020/ *Geriatric Nursing*	Quanti-qualitativa/ Projeto convergente de métodos mistos paralelos	Avaliar a relação entre uma intervenção de Prática Colaborativa Interprofissional (IPCP) para idosos residentes na comunidade, *Geriatric Outreach e Training with Care!* ( *GOT Care!* ), e a redução de visitas ao Departamento de Emergência (DE) para os 51 idosos participantes.	P ^†^: Pessoas com idade maior ou igual a 65 anos residentes na comunidade identificadas como de alto risco, devido à alta utilização de serviços de emergência ou polifarmácia com múltiplas condições. C ^‡^: O estudo utilizou os conceitos de variáveis do cliente (fisiológicas, psicológicas, socioculturais, espirituais e de desenvolvimento), estressores, linhas de defesa, linhas de resistência e estratégias de prevenção primária, secundária e terciária. C ^§^: Serviço de saúde comunitário.
Larijani F, Fotokian Z, Jahanshahi M, Tabi SR ^(^ ^24^ ^)^ .	Irã/2021/ *Nursing and Midwifery Studies*	Quantitativa	Avaliar o efeito do modelo de sistemas de Neuman na ansiedade de idosos à espera de colonoscopia.	P ^†^: Pessoas com idade maior ou igual a 60 anos fluentes em língua persa, que não receberam drogas ansiolíticas ou antipsicóticas nos últimos seis meses, sem deficiências auditivas ou cognitivas, sem histórico de estresse grave nos últimos três meses, sem histórico de endoscopia ou colonoscopia, e que obtiveram, pelo menos, 50% da pontuação total na lista de verificação de avaliação de necessidades do Modelo de Sistemas de Neuman. C ^‡^: O estudo explorou os conceitos de variáveis do cliente (fisiológicas, psicológicas, socioculturais e de desenvolvimento), estressores intrapessoais, interpessoais e extrapessoais potenciais e reais, definição de metas, intervenções de enfermagem e avaliação, conforme o processo de enfermagem sustentados pelo Modelo de Sistemas de Neuman. C ^§^: Atenção à saúde domiciliar.
Pereira F, Bieri M, Martins MM, Del Río Carral M, Verloo H ^(^ ^25^ ^)^ .	Itália/ 2022/ *Nursing Reports*	Qualitativa/ Estudo descritivo	Identificar e categorizar os estressores vivenciados e as estratégias de reconstituição adotadas por idosos, seus cuidadores informais e profissionais de saúde no manejo medicamentoso de idosos após a alta hospitalar.	P ^†^: Pessoas com idade maior ou igual a 65 anos hospitalizadas nos últimos 90 dias gerenciando, pelo menos, cinco medicamentos diferentes diariamente. C ^‡^: O estudo explorou os conceitos de fatores estressores extrapessoais e as estratégias de reconstituição extrapessoais adotadas por idosos, seus cuidadores informais e profissionais de saúde no manejo medicamentoso após alta hospitalar. C ^§^: Atenção à saúde domiciliar.

* PCC = População, Conceito e Contexto;

† P = População;

‡ C = Conceito;

§ C = Contexto

Dos 14 estudos selecionados, constatou-se que a prevalência de publicações foi maior em 2021, representando 14,2% (n=2) da amostra. Por outro lado, os outros 12 estudos foram distribuídos em diferentes anos de publicação, com cada ano representando, aproximadamente, 7,1% da amostra, representado por um único estudo (n=1).

Quanto à procedência dos estudos, destacou-se que 50% (n=7) foram provenientes dos Estados Unidos da América (EUA), enquanto 14,2% (n=2) foram realizados em Portugal e no Brasil. A abordagem metodológica que prevaleceu foi a qualitativa 71,4% (n=10), seguida da quantitativa 21,4%(n=3) e quanti-qualitativa 7,1% (n=1).

## Discussão

Os estudos selecionados foram organizados em duas categorias, considerando a similaridade de seu conteúdo. A primeira categoria aborda a implementação do Modelo de Sistemas de Neuman nos contextos de cuidado hospitalares, institucionais e ambulatoriais; e a segunda categoria envolve a utilização do Modelo de Sistemas de Neuman nos contextos comunitário e domiciliar.

### Categoria 1 - Implementação do Modelo de Sistemas de Neuman em ambientes hospitalares, institucionais e ambulatoriais

 Sete estudos exploraram a aplicabilidade do Modelo de Sistemas de Neuman em contextos hospitalares, institucionais e ambulatoriais ^(^ ^12^ ^-^ ^18^ ^)^ . Essas investigações abordaram condições específicas, tais como: atenção à pessoa idosa com câncer assistida no serviço de atenção hospitalar ^(^ ^12^ ^)^ ; cuidado da pessoa idosa com artrite reumatoide atendida em serviço de atenção hospitalar ^(^ ^13^ ^)^ ; práticas de toque de conforto em casas de repouso de médio porte ^(^ ^14^ ^)^ ; estímulo à espiritualidade em casas de repouso e unidades de cuidado de enfermagem ^(^ ^25^ ^)^ ; prevenção de lesões por pressão na unidade de terapia intensiva de um hospital ^(^ ^16^ ^)^ ; e a identificação e gestão de estressores durante a hospitalização, incluindo clínica médica e clínica cirúrgica ^(^ ^17^ ^-^ ^18^ ^)^ . 

 A abordagem do Modelo de Sistemas se mostrou efetiva ao lidar com os estressores decorrentes dos efeitos da colostomia na atenção à idosa com câncer, abrangendo diferentes dimensões. No âmbito intrapessoal, a alteração dos hábitos intestinais desencadeou estressores psicológicos, resultando em desafios emocionais e psicológicos que impactaram a capacidade de adaptação da idosa. Na esfera interpessoal, a ausência de suporte social gerou um impacto negativo adicional, dificultando, ainda mais, o processo de adaptação. Além disso, a hospitalização em si representou um estressor na dimensão extrapessoal, afetando as interações sociais da paciente. As intervenções de enfermagem foram direcionadas para fortalecer as linhas de defesa da idosa, fornecendo apoio adaptativo e buscando manter seu bem-estar ideal ^(^ ^12^ ^)^ . 

 A necessidade de explorar de forma aprofundada as crenças e mecanismos de enfrentamento do câncer, evidenciada no estudo da idosa com colostomia, é enfatizada na eficácia da implementação do Modelo de Sistemas de Neuman em pacientes com artrite reumatoide. A aplicação desse modelo às pessoas idosas com artrite reumatoide demonstrou ser eficaz ao considerar de maneira abrangente os impactos sociais, emocionais e físicos dessa condição crônica, incorporando elementos como espiritualidade e capacidade de adaptação. Ao analisar os efeitos combinados desses impactos na percepção geral de saúde das pessoas idosas, destacou-se a importância da espiritualidade no enfrentamento das crises agudas da doença. Além disso, a utilização do modelo promoveu uma colaboração mais ampla entre pacientes e cuidadores, oferecendo alívio e soluções para suas complexas necessidades de saúde ^(^ ^13^ ^)^ . 

 Verificaram-se, também, efeitos benéficos do Modelo de Sistemas de Neuman, aliados ao toque de conforto para autoestima, bem-estar, processos sociais, estado de saúde, satisfação com a vida, autorrealização, fé ou crença e autorresponsabilidade de mulheres idosas assistidas em instituições de cuidados de longa duração ^(^ ^14^ ^)^ . 

 Os resultados do estudo sobre o toque de conforto evidenciaram impacto significativo nas percepções das pessoas idosas, promovendo melhorias no bem-estar, autoestima e outras variáveis avaliadas positivamente. Esses benefícios se alinham aos achados do estudo que utilizou o Modelo de Sistemas na prevenção de lesão por pressão em pacientes internados, ressaltando a eficácia de abordagens que consideram não apenas fatores físicos, mas também aspectos emocionais, sociais e ambientais para aprimorar a saúde e o bem-estar dos pacientes. A aplicação combinada do Modelo de Sistemas com a escala de avaliação de risco para lesão por pressão demonstrou, desta maneira, eficácia na prevenção primária, permitindo à equipe de enfermagem adotar medidas preventivas específicas para reduzir estressores potenciais e fortalecer as defesas dos pacientes contra o estresse ^(^ ^16^ ^)^ . 

 Em outra pesquisa, destacou-se a relevância da educação em saúde para oferecer cuidados holísticos, considerando a espiritualidade de idosos com base no Modelo de Neuman. Realizado em casas de repouso e unidades de cuidado de enfermagem, o estudo revelou efeitos positivos na saúde mental e física, fortalecendo as relações interpessoais e a capacidade de enfrentar desafios. Notou-se que, à medida que a saúde dos idosos se deteriora, a dependência espiritual aumenta, indicando o desejo de receber cuidados espirituais dos profissionais de saúde ^(^ ^15^ ^)^ . 

 A utilização do Modelo de Sistemas de Neuman para identificação e gestão de estressores durante a hospitalização de pessoas idosas também evidenciou que determinados estressores afetam diversas áreas clínicas e pessoais, tais como: idade avançada, capacidade física reduzida, conhecimento limitado e emoções negativas, além de influenciarem relacionamentos familiares, privacidade, autonomia e o ambiente hospitalar em si ^(^ ^17^ ^)^ . Tais descobertas têm embasado intervenções de enfermagem focalizadas na síndrome do idoso frágil, distúrbios do sono, ansiedade, medo e sofrimento espiritual, visando mitigar o impacto desses estressores e aprimorar a experiência dos idosos durante sua permanência no hospital ^(^ ^18^ ^)^ . 

Os achados desses estudos ressaltam a eficácia das intervenções terapêuticas em ambientes hospitalares, institucionais e ambulatoriais na melhoria da autoestima e bem-estar dos pacientes. A aplicação do Modelo de Sistemas de Neuman revelou um impacto significativo na qualidade de vida das pessoas idosas, abrangendo dimensões físicas, psicológicas, sociais e espirituais. A abordagem da espiritualidade demonstra ser um elemento fundamental nas intervenções de saúde, influenciando a percepção de propósito de vida e a capacidade de enfrentamento de estressores. Destaca-se a importância da interação entre pessoas idosas, família e profissionais de saúde para promover vínculos afetivos e de confiança, fortalecendo suas linhas de defesa e resistência, resultando em intervenções eficazes com impacto positivo em suas vidas.

### Categoria 2 - Utilização do Modelo de Sistemas de Neuman em ambientes comunitário e domiciliar

 Essa categoria abarca sete estudos ^(^ ^19^ ^-^ ^25^ ^)^ , os quais exploraram a aplicação do Modelo de Sistemas de Neuman nos ambientes comunitário e domiciliar enfocando o cuidado para a promoção da saúde de pessoas idosas por meio do(a): assistência domiciliar a idosos após um infarto agudo do miocárdio ^(^ ^19^ ^)^ ; suporte domiciliar pós-alta hospitalar após um episódio de acidente vascular cerebral(AVC) ^(^ ^20^ ^)^ ; atenção a idosos com Parkinson assistidos pelo serviço de saúde comunitário ^(^ ^21^ ^)^ ; de atividades de educação em saúde realizadas pelo serviço de saúde comunitário ^(^ ^22^ ^)^ ; intervenção de Prática Colaborativa Interprofissional (IPCP) para idosos residentes na comunidade ^(^ ^23^ ^)^ ; redução da ansiedade em idosos que aguardam colonoscopia no domicílio ^(^ ^24^ ^)^ ; e gestão medicamentosa após a alta hospitalar ^(^ ^25^ ^)^ . 

O Modelo de Sistemas de Neuman se mostrou aplicável aos ambientes comunitário e domiciliar, abarcando a família, a comunidade e o domicílio. Ele permitiu uma avaliação abrangente das necessidades e recursos das pessoas e famílias, possibilitando a implementação de intervenções preventivas, curativas e de reabilitação. Além disso, o modelo valorizou a participação ativa dos indivíduos e das famílias no cuidado à saúde, fortalecendo o vínculo entre os profissionais de saúde e a comunidade.

 Este modelo tem exibido efetividade na recuperação domiciliar do idoso após sofrer um infarto agudo do miocárdio, ao promover seu bem-estar alinhado com seus valores pessoais, estabelecendo metas progressivas e utilizando adequadamente os recursos disponíveis ^(^ ^18^ ^)^ . 

 Assim, a aplicação do Modelo de Sistemas de Neuman, na prevenção terciária evidenciou melhorias significativas na restauração do equilíbrio do sistema cliente após um infarto agudo do miocárdio, refletindo resultados semelhantes em um estudo sobre ações preventivas direcionadas à recuperação de idosos após alta hospitalar subsequente a um episódio de AVC. O estudo permitiu identificar estressores individuais e familiares, como: privação de sono, ansiedade, dificuldades comunicativas e falta de apoio externo, com um plano de cuidado focado na promoção da autonomia, otimização dos padrões de sono e implementação de ajustes ambientais para facilitar a recuperação. Essas estratégias incluíram intervenções motivacionais, educacionais e comportamentais tanto no âmbito individual quanto no contexto familiar, destacando a adaptabilidade e eficácia do modelo em diferentes cenários clínicos ^(^ ^19^ ^)^ . 

 A aplicação do Modelo de Sistemas de Neuman permitiu ao enfermeiro estabelecer metas efetivas de cuidado por meio de intervenções de prevenção primária, secundária e terciária; estas foram alinhadas com as necessidades de saúde em curto, médio e longo prazos de um casal de idosos, no qual um dos parceiros foi diagnosticado com a doença de Parkinson. Essas metas abrangeram diferentes aspectos, como o apoio emocional, o manejo da incontinência noturna e o enfrentamento dos medos do idoso em relação à perda da independência. Por meio de uma comunicação eficiente entre os profissionais de saúde envolvidos, foi possível garantir a continuidade do cuidado e oferecer o suporte necessário, resultando em melhoria na qualidade do sono do idoso e a redução do isolamento social da esposa, que desempenhava o papel de cuidadora ^(^ ^20^ ^)^ . 

 Outro ponto crucial foi a utilização do Modelo de Sistemas de Neuman como uma sólida ferramenta longitudinal de cuidado por meio do serviço de saúde comunitário. As ações de educação em saúde proporcionaram melhorias significativas no bem-estar das pessoas idosas, capacitando-as com conhecimentos sobre medidas preventivas, incentivando mudanças nos estilos de vida e promovendo o desenvolvimento de redes de apoio. Esses impactos foram especialmente notáveis entre idosos de baixa renda, resultando na promoção de sua saúde e na melhoria da qualidade de vida ^(^ ^21^ ^)^ . 

 Dessa forma, constata-se que a implementação do Modelo de Sistemas de Neuman na atenção comunitária desempenha um papel crucial no bem-estar das pessoas idosas. Estudo conduzido no serviço de saúde comunitário identificou a contribuição dos fatores estressores que impactam a qualidade de vida dos idosos, seguida do desenvolvimento de estratégias de prevenção primária, secundária e terciária, para fortalecer as linhas de defesa e resistência das pessoas idosas e promover o bem-estar das mesmas ^(^ ^23^ ^)^ . 

 Verificaram-se, também, resultados positivos na redução da ansiedade em idosos que aguardam uma colonoscopia no domicílio, por meio de intervenções específicas direcionadas a cada estressor identificado, tais como preparação intestinal, problemas de saúde, distúrbios do sono, medo do diagnóstico, falta de conhecimento e isolamento social ^(^ ^24^ ^)^ . 

 Quanto à atenção à gestão medicamentosa no domicílio após a alta hospitalar de pessoas idosas, a identificação dos estressores relacionados a esse processo destacou problemas de comunicação, colaboração e coordenação deste cuidado entre idosos, cuidadores informais e profissionais de saúde. A falta de participação das pessoas idosas nas decisões sobre a medicação levantou preocupações, resultando em dificuldades de compreensão e adesão ao tratamento. Nesse contexto, ressalta-se a importância de uma abordagem centrada no paciente, interprofissional e colaborativa para aprimorar a segurança na gestão medicamentosa ^(^ ^25^ ^)^ . 

Os estudos apresentados confirmam o papel crucial da enfermagem na promoção da saúde dos idosos, fortalecendo suas redes de apoio e implementando estratégias de prevenção primária, secundária e terciária em contextos comunitários e domiciliares, visando manter o bem-estar e o equilíbrio do sistema do cliente.

Quanto às lacunas do conhecimento evidenciadas, observou-se a falta de propostas participativas, horizontais e intergeracionais para o enfrentamento eficaz de estressores no curso da vida considerando o envelhecimento saudável. Além disso, o estudo evidenciou a carência de investigações do papel do enfermeiro como membro de equipes multidisciplinares, concentrando-se na identificação de estressores e na implementação de intervenções adequadas nos diferentes contextos de cuidado, visando promover o envelhecimento saudável e enfrentamento eficaz de estressores ao longo da vida.

Este estudo enfrentou algumas limitações, incluindo a inacessibilidade a três publicações integrais, restringindo a possibilidade de uma análise profunda destes documentos. Adicionalmente, outras limitações foram evidenciadas. A estratégia de busca empregada pode não ter abrangido todos os termos relevantes, particularmente, considerando o contexto “serviços de saúde”, uma vez que este termo apresenta uma ampla variação terminológica entre diferentes nações. Expandir a revisão para outras bases de dados relevantes, também, poderia ter ampliado a abrangência do estudo, permitindo a incorporação de pesquisas adicionais sobre a temática proposta.

## Conclusão

Verificou-se que a integração da abordagem de sistemas de Neuman, na prática de enfermagem gerontológica, em diversos cenários de cuidado, como ambientes hospitalares, institucionais, ambulatoriais, comunitários e domiciliares, revelou eficácia e adaptabilidade ao considerar as pessoas idosas como indivíduos abrangentes e multidimensionais.

Essa abordagem atende às diversas necessidades das pessoas idosas ao considerar as cinco variáveis do cliente: fisiológicas; psicológicas; espirituais; socioculturais; e de desenvolvimento, implementando ações de cuidado para fortalecer as linhas de defesa e resistência do cliente, além de reconhecer os fatores estressores potenciais e reais do cliente (intrapessoais, interpessoais e extrapessoais), utilizando estratégias de intervenção primária, secundária e terciária para aprimorar seu bem-estar e qualidade de vida.

O desdobramento desta pesquisa implica o desenvolvimento de estudos direcionados à promoção do envelhecimento saudável, considerando os elementos conceituais da teoria e as complexidades inerentes à senescência, com o intuito de aprimorar as práticas de enfermagem em benefício do bem-estar da população que envelhece.
